# The palmitoylation of AEG-1 dynamically modulates the progression of hepatocellular carcinoma

**DOI:** 10.7150/thno.78377

**Published:** 2022-10-03

**Authors:** Binhui Zhou, Ying Wang, Lichen Zhang, Xiaoyi Shi, Hesheng Kong, Mengjie Zhang, Yang Liu, Xia Shao, Zhilong Liu, Hongxu Song, Wushan Li, Xiaoxi Gao, Yanli Chang, Chenzhuo Dou, Wenzhi Guo, Shuijun Zhang, Xiaohong Kang, Jie Gao, Yinming Liang, Junfeng Zheng, Eryan Kong

**Affiliations:** 1 Laboratory of Mouse Genetics, Institute of Psychiatry and Neuroscience, Xinxiang Medical University, Xinxiang 453003, Henan, China.; 2 Laboratory of Genetic Regulators in the Immune System, Henan Collaborative Innovation Center of Molecular Diagnosis and Laboratory Medicine, Xinxiang Medical University, Xinxiang 453003, Henan, China.; 3 Henan Key Laboratory of Immunology and Targeted Therapy, School of Laboratory Medicine, Xinxiang Medical University, Xinxiang 453003, Henan, China.; 4 Department of Hepatobiliary and Pancreatic Surgery, The First Affiliated Hospital of Zhengzhou University, Zhengzhou 450052, Henan, China.; 5 Department of Oncology, the First Affiliated Hospital of Xinxiang Medical University, Xinxiang, China.

**Keywords:** protein palmitoylation, AEG-1, SND1, hepatocellular carcinoma, HCQ

## Abstract

**Rationale:** Protein palmitoylation is tightly related to tumorigenesis or tumor progression as many oncogenes or tumor suppressors are palmitoylated. AEG-1, an oncogene, is commonly elevated in a variety of human malignancies, including hepatocellular carcinoma (HCC). Although AEG-1 was suggested to be potentially modified by protein palmitoylation, the regulatory roles of AEG-1 palmitoylation in tumor progression of HCC has not been explored.

**Methods:** Techniques as Acyl-RAC assay and point mutation were used to confirm that AEG-1 is indeed palmitoylated. Moreover, biochemical experiments and immunofluorescent microscopy were applied to examine the cellular functions of AEG-1 palmitoylation in several cell lines. Remarkably, genetically modified knock-in (AEG-1-C75A) and knockout (Zdhhc6-KO) mice were established and subjected to the treatment of DEN to induce the HCC mice model, through which the roles of AEG-1 palmitoylation in HCC is directly addressed. Last, HCQ, a chemical compound, was introduced to prove in principal that elevating the level of AEG-1 palmitoylation might benefit the treatment of HCC in xenograft mouse model.

**Results:** We showed that AEG-1 undergoes palmitoylation on a conserved cysteine residue, Cys-75. Blocking AEG-1 palmitoylation exacerbates the progression of DEN-induced HCC *in vivo*. Moreover, it was demonstrated that AEG-1 palmitoylation is dynamically regulated by zDHHC6 and PPT1/2. Accordingly, suppressing the level of AEG-1 palmitoylation by the deletion of Zdhhc6 reproduces the enhanced tumor-progression phenotype in DEN-induced HCC mouse model. Mechanistically, we showed that AEG-1 palmitoylation adversely regulates its protein stability and weakens AEG-1 and staphylococcal nuclease and tudor domain containing 1 (SND1) interaction, which might contribute to the alterations of the RISC activity and the expression of tumor suppressors. For intervention, HCQ, an inhibitor of PPT1, was applied to augment the level of AEG-1 palmitoylation, which retards the tumor growth of HCC in xenograft model.

**Conclusion:** Our study suggests an unknown mechanism that AEG-1 palmitoylation dynamically manipulates HCC progression and pinpoints that raising AEG-1 palmitoylation might confer beneficial effect on the treatment of HCC.

## Introduction

AEG-1 is a multifunctional oncogene that is overexpressed in a wide variety of human malignancies, including HCC 1-5. The overexpression of AEG-1 or SND1, an AEG-1 interacting protein, in liver cancer cells significantly enhances the activity of RNA-induced silencing complex (RISC), which may increase the degradation of tumor suppressor messenger RNAs e.g. PTEN, CDKN1A (p21), SPRY2, and TGFBR2 and therefore promotes the occurrence and development of HCC 6. Clinically, it was further suggested that enhanced expression of AEG-1 is a marker of poor prognosis in cancer patients, emphasizing the scenario that understanding how the amount of protein expression regulated might be the most valuable factor to target tumorigenesis due to AEG-1 overload. Indeed, a recent study reported that FBXW7-mediated AEG-1 ubiquitination significantly suppresses breast cancer cell proliferation by attenuating AEG-1 protein stability 7. Considering the facts that ubiquitination often cross talk with other post-translational modifications (PTM) 8, 9, we wondered if other PTM, as protein S-palmitoylation (hereinafter referred to as palmitoylation), might occur on AEG-1 and thus involved in the regulation of AEG-1 stability and function.

Intriguingly, AEG-1 was identified as a candidate protein of palmitoylation in several palm-proteomics studies 10-12. Palmitoylation is one of the most common and reversible lipid post-translational modifications, where a 16-carbon palmitoyl group is added onto cysteine residue via thioester linkage 13. Remarkably, palmitoylation is involved in regulating many aspects of modified protein e.g. subcellular localization, protein-protein interaction, and protein stability 14, 15. To date, 23 mammalian palmitoyl-transferases, namely zDHHCs 16 and 5 depalmitoylating enzymes including APT1/2, PPT1/2 and ABHD17a 17-19 have been identified. The link between palmitoylation and various types of human malignancies has been firmly established by the findings that many oncogenes and tumor suppressors are palmitoylated 8, 20-23, yet, such relationship is labile in HCC.

Here, we showed that AEG-1 is generally palmitoylated in several cell lines including HCC cancer cells, furthermore, such palmitoylation on AEG-1 is reversible, managed by zDHHC6 mediated palmitoylation and PPT1/2 mediated depalmitoylation. Surprisingly, AEG-1 palmitoylation negatively manipulates its protein stability and the assembly with SND1 for RISC activity, through which the hepatocellular carcinogenesis is modulated. Accordingly, HCQ, a suggested inhibitor of PPT1 24, was applied for enhancing AEG-1 palmitoylation and its degradation, and ultimately inhibiting HCC progression. Our findings strengthened the understanding of protein palmitoylation in HCC and moreover, pinpointed that AEG-1 palmitoylation might be a valuable therapeutic target for treating HCC.

## Methods

### Antibodies, reagents and plasmids

The antibody against MYC (AE070) was purchased from ABclonal, and PPT2 (YN3889) was purchased from Immunoway. The HA (H3663), FLAG (F7425 for western blotting and F1804 for immunofluorescence) and zDHHC6 (PA5-113325) antibodies were purchased from Sigma-Aldrich, and AEG-1 (ab205646), FBXW7 (ab192328), PPT1 (ab135516) and β-tubulin (ab6046) were purchased from Abcam. Secondary antibodies goat anti-mouse IgG H&L (HRP) (ab6789) and goat anti-rabbit IgG H&L (HRP) (ab6721) were purchased from Abcam, goat anti-mouse Alexa Fluor 488 (R37120), goat anti-rabbit Alexa Fluor 594 (R37117) and goat anti-rabbit Alexa Fluor 647 (A21245) were purchased from Thermo Fisher Scientific. 2-Bromopalmitate (2-BP, 238422), Palmostatin B (Palm B, 178501), Hydroxylamine hydrochloride (HAM, 431362), N-Ethylmaleimide (NEM, 04259), Chloroquine (CQ, C6628), Hydroxychloroquine (HCQ, PHR1782), Diethylnitrosamine (DEN, N0258), Collagenase IV (DN25), and DNase I (C5138) were purchased from Sigma-Aldrich. Thiopropyl Sepharose 6B (17042001), MG132 (S2619) and Cycloheximide (CHX, HY-12320) were purchased from GE Healthcare, Selleck and MCE, respectively. For expression plasmids, wildtype AEG-1 (AEG-1-WT) and the mutant AEG-1 (AEG-1-MT, the cysteine ​​at position 75 was mutated to alanine) were respectively subcloned in pCMV3-C-HA and pCMV3-C-MYC. FBXW7 and Lamp1 were subcloned in pCMV3-C-Flag and pCMV3-N-RFP, respectively. Five depalmitoylating enzymes including APT1/2, PPT1/2 and ABHD17a were respectively subcloned in pCMV3-C-Flag, while palmitoyl-transferase zDHHC6 was subcloned in pCMV3-C-HA. Membrane marker plasmid mTagRFP-Membrane-1 was purchased from Addgene. For generation of PPT1, PPT2, zDHHC2, zDHHC5, zDHHC6 and zDHHC24 knockout cell lines of HEK293T, guide RNA (gRNA) sequences of six human genes for CRISPR/Cas9 which listed in Supplementary Table 1 were designed at CRISPR design website (http://crispr.mit.edu/) 25. The gRNAs target the exon 5, 2, 4, 4, 3 and 2 of *PPT1*, *PPT2*, *zDHHC2*, *zDHHC5*, *zDHHC6* and *zDHHC24* genes, respectively. The complementary oligonucleotides for gRNAs were annealed, and cloned into pX459 CRISPR/Cas9-Puro vector (Addgene). Clone identity was confirmed by sequencing (Invitrogen, Shanghai, China).

### Cell culture and transfection

The cell lines HEK293T, THLE-3, Huh7 and HepG2 were obtained from the American type culture collection. The HEK293T, Huh7 and HepG2 cells were cultured in DMEM medium supplemented with 10 % fetal bovine serum (FBS, PAN), THLE-3 cells were cultured in RPMI1640 medium supplemented with 10 % FBS. All of the cell culture media contained 100 mg/mL streptomycin and 100 U/mL penicillin. The cells were incubated in 5 % CO_2_ at 37 ℃. Lipofectamine™ 3000 transfection reagent (L3000015) was purchased from Thermo Fisher Scientific and utilized for cell transfection as indicated by the manufacturer's instruction.

### Acyl-RAC assay

Acyl-RAC assay was performed as previously described 26. Briefly, cells were collected for protein extraction, then equal amounts of protein (1 mg) were blocked by NEM at 50 ℃ for 1 h with frequent vortexing. Following cold acetone precipitation, the pellet was washed with 70 % acetone for three times and resuspended in 640 μl binding buffer, approximately 40 μl of each sample was saved as the “total input,” and the remaining resuspension was divided into two parts. Each one added 40 μl prewashed Thiopropyl Sepharose and 40 μl 2 M HAM (freshly prepared, pH 7.4) or 2 M NaCl. Binding reactions were carried out on a rotator at room temperature for 4 h. Resins were washed five times with binding buffer and then eluted by 50 μl of 1 × SDS-PAGE loading buffer, boiled at 100 ℃ for 5 min and then subjected to western blotting analyses.

### Western blotting

Cells or tissues were lysed with RIPA lysis buffer containing protease inhibitor for protein extraction. Standard western blotting assay was performed to analyze protein expression. The indicated primary antibodies were used for assay, and membrane was detected by using chemiluminescent HRP substrate (Millipore) in automatic chemiluminescence image analysis system (Tanon-5200). The intensity of bands were quantified using Image J software.

### 2-BP, Palm B and HCQ incubation

For 2-BP treatment, HEK293T or Huh7 cells were respectively cultured with 100 μM 2-BP for the designed time point, or vehicle alone. For Palm B treatment, Huh7 cells were cultured with 50 μM Palm B for the designed time point, or vehicle alone. For HCQ treatment, HepG2 cells were respectively cultured with 20 μM HCQ or vehicle alone for 12 h. Then whole-cell lysates were prepared and then Acyl-RAC and western blotting were performed as described above for detecting AEG-1 palmitoylation level.

### Cell proliferation assay

Huh7 and HepG2 cells were seeded in 96-well plates at a density of 1 × 10^4^ cells per well. After 24 h, cells were respectively transfected with designed plasmids, or treated with 50 μM Palm B, 20 μM HCQ or vehicle alone. Cell proliferation was evaluated using the Cell Counting Kit-8 (CCK-8, Kemix, KC0301) for the designed time point.

### Immunofluorescence staining

Huh7 cells were seeded on coverslips (NEST) and transfected with the desired plasmids. Next, cells were fixed with 4 % paraformaldehyde and incubated with 0.1 % Triton X-100 (for cytoplasm protein detection) or without Triton X-100 treatment (for membrane protein detection). After blocked with 3 % BSA and incubated with primary antibodies overnight at 4 ℃, cells were then incubated with fluorescence-conjugated secondary antibodies and subsequently counterstained with DAPI. Images were taken using a Leica TCS SP8 confocal microscopy.

### Generation of AEG-1 point mutation and Zdhhc6^-/-^ mice and hepatocellular carcinoma induction

C57BL/6 (B6) mice were purchased from Beijing Vital River Laboratory Animal Technology Co., Ltd. AEG-1 point mutation and Zdhhc6^-/-^ mice were generated as described previously 26, 27. The sequences of the primer pairs were listed in Supplementary Table 4. For hepatocellular carcinoma induction, 14-day-old male mice were injected intraperitoneally (i.p.) with DEN (25 μg/g) and then fed with regular chow diet and water until sacrificed (9 months). The animal experiments were performed according to guidelines approved by the committee on animal care at Xinxiang Medical University.

### Generation of knockout cell lines using CRISPR/Cas9

HEK293T cells were co-transfected with PPT1/gRNA #1 and #2, PPT2/gRNA #1 and #2, zDHHC2/gRNA #1 and #2, zDHHC5/gRNA #1 and #2, zDHHC6/gRNA #1 and #2 or zDHHC24/gRNA #1 and #2 as described above, respectively. Two days after transfection, single cell was sorted by flow cytometry and cultured until colonies were formed. Individual colonies were picked and screened by PCR, agarose gel electrophoresis and DNA sequencing detection, the sequences of the primers were listed in Supplementary Table 2. Positive clones were used for the current studies.

### APEGS assay

APEGS assay was performed as previously described 28. Briefly, WT and zDHHC6^-/-^ HEK293T cells were cultured and transfected with AEG-1-WT-Myc for 24 h, and then cells were collected for protein extraction. Equal amounts of protein (500 μg) were reduced with 25 mM TCEP at 55 ℃, 100 rpm for 1 h, and free cysteine residues were blocked with 50 mM NEM at 50 ℃, 100 rpm for 3 h. After cold acetone precipitation, the pellet was washed with 70 % acetone for three times and resuspended in 100 μl 1 × PBS with 4 % SDS and 5 mM EDTA, then 900 μl 1 M HAM (freshly prepared, pH 7.4) or 1 M Tris-HCl was added and incubated at 37 ℃, 100 rpm for 1 h. After cold acetone precipitation and 70 % acetone washing three times, proteins were resuspended in 1 ml 1 × PBS with 4 % SDS were PEGylated with 10 mM mPEG-2k at 25 ℃, 100 rpm for 1 h. 20 mM NEM was used as a negative control. After cold acetone precipitation, the pellet was resuspended with 40 μl of 1 × SDS-PAGE loading buffer, boiled at 100 ℃ for 5 min and then subjected to western blotting analyses.

### RNA extraction and qRT-PCR

Total RNA was extracted from cell lines and mice liver tissues using the TRIzol reagent (Invitrogen). cDNA synthesis and qRT-PCR were performed to quantify mRNA levels in cell lines and mice livers. The sequences of the primers used throughout this study were listed in Supplementary Table 3.

### Co-immunoprecipitation (co-IP)

For co-IP assays, cells were lysed with RIPA buffer containing protease inhibitors. The target protein was pulled down with protein A/G magnetic beads (HY-K0202, MCE) from whole-cell lysates by incubating with desired antibodies. Beads were washed with RIPA lysis buffer five times, and bound protein was eluted by 1 × SDS-PAGE loading buffer, and subjected to western blotting analysis.

### Patient information and tissue microarray immunohistochemistry (IHC)

This study conducted on 133 pairs of paraffin-embedded samples collected from HCC tumor tissue and adjacent normal tissue histopathologically and clinically diagnosed at the First Affiliated Hospital of Zhengzhou University from January 2012 to September 2019. The study was approved by the ethics committee of our department, and each patient signed a written informed consent. IHC of zDHHC6 was performed as described previously 29. The results of tissue microarray IHC were evaluated and scored by calculating the H-score and the percentage of zDHHC6 positive area. H-score = percentage of weak intensity cells × 1 + percentage of moderate intensity cells × 2 + percentage of strong intensity cells × 3.

### AEG-1 degradation assay

Cycloheximide (CHX) experiments were performed with a minor modification as described previously 30. Briefly, the HEK293T cells were individually transfected with the designed plasmids and with or without HCQ treatment, respectively. After 24 h, the cells were treated with 100 μg/ml CHX for 0, 8 and 12 h. In addition, to determine the pathway for AEG-1 degradation, the pCMV-AEG-1-WT transfected cells were treated with DMSO, 100 μg/ml CHX, 100 μg/ml CHX plus 10 μM MG132 and 100 μg/ml CHX plus 100 μM CQ for 12 h, respectively. Whole cell extracts were produced and equal amounts of total protein were subjected to western blotting analysis.

### *In vivo* ubiquitination assay

HEK293T cells were transfected with the desired plasmids, including expressing FBXW7-Flag, AEG-1-WT-Myc or AEG-1-MT-Myc with or without HA-Ub, respectively, for 24 h. Then the cells were treated with 10 µM MG132 for 6 h and harvested for protein extraction. The levels of AEG-1-WT-Myc and AEG-1-MT-Myc ubiquitination was determined by IP followed by western blotting assay.

### Flow cytometry analysis

The mice were anesthetized by intraperitoneal injection of anesthetic, and the heart was perfused with normal saline to flush out the blood. The livers were collected from normal and DEN-induced HCC mice and dissociated in RPMI 1640 medium supplemented with 10 % FBS, 0.2 mg/ml Collagenase IV and 0.1 mg/ml DNase I with the gentleMACS™ Octo Dissociator, and then incubated at 37 ℃, 200 rpm for 25 min, respectively. Then all samples were immediately added with 5 ml FACS buffer to terminate the digestion reaction, and respectively filtered into 15 ml tubes with 30 μm MACS SmartStrainer to obtain single cell suspension. The cells were collected by centrifugation and resuspended with 5 ml FACS buffer, 10 μl cells were used for CD45-FITC (MABF320, Millipore) staining and counting. Then 0.5 million CD45^+^ cells were respectively stained with antibody mixture. Afterward, all the samples were analyzed using an Attune NxT or BD FACSCanto II using FlowJo 10.0 software (BD Biosciences).

### RISC activity detection

The RISC activity detection assay was performed as described previously 6. Briefly, miRNA23 was subcloned in pmirGLO vector which named pmirGLO 1 ×. Then Huh7 cells were seeded in 96-well plate at a density of 1 × 10^4^ cells per well. After 24 h, cells were respectively transfected with a mixture containing pmirGLO 1 ×, AEG-1-WT or AEG-1-MT, and control sdRNA or miR-23 sdRNA for 36 h. Thereafter, the transfected cells were lysed and used to RLUC and FLUC activity detection by using Firefly & Renilla Dual Luciferase Assay Kit (F6075, US EVERBRIGHT^®^ INC.).

### Subcutaneous tumor‑bearing nude mouse experiment

8-week old male Balb/c nude mice were purchased from Vital River (https://vitalriver.biomart.cn/). Huh7 cells (2 × 10^6^) in 0.1 ml of 1 × PBS were injected subcutaneously into the right flank of Balb/c nude mice. When tumor volume reached approximately 50 mm^3^ (14 days after implantation), mice were randomly divided into two groups, respectively (n=6 per group): PBS group and HCQ group. The dose of HCQ was 50 μg/g, intraperitoneal injection, for 39 consecutive days. Tumor length (a) and width (b) were measured using a vernier caliper every 3 days, and then tumor volume was calculated using the formula: 1/2ab^2^. The mice were sacrificed on the 53 days after implantation, and tumors were isolated and weighed.

### Statistical analysis

Kaplan-Meier analysis and log-rank test were used to compare overall survival and recurrence free survival in different groups. All the other data were shown as mean ± standard error (SE). Differences were analyzed by two-tailed Student's *t*-test between two groups and one-way ANOVA with Dunnett's multiple comparison test for comparison among three groups: *, *p* < 0.05; **, *p* < 0.01; ***, *p* < 0.001; ****, *p* < 0.0001, ns indicates no significance.

## Results

### AEG-1 is a palmitoylated protein and might be involved in regulating liver cancer cell proliferation

**Figure 1:**
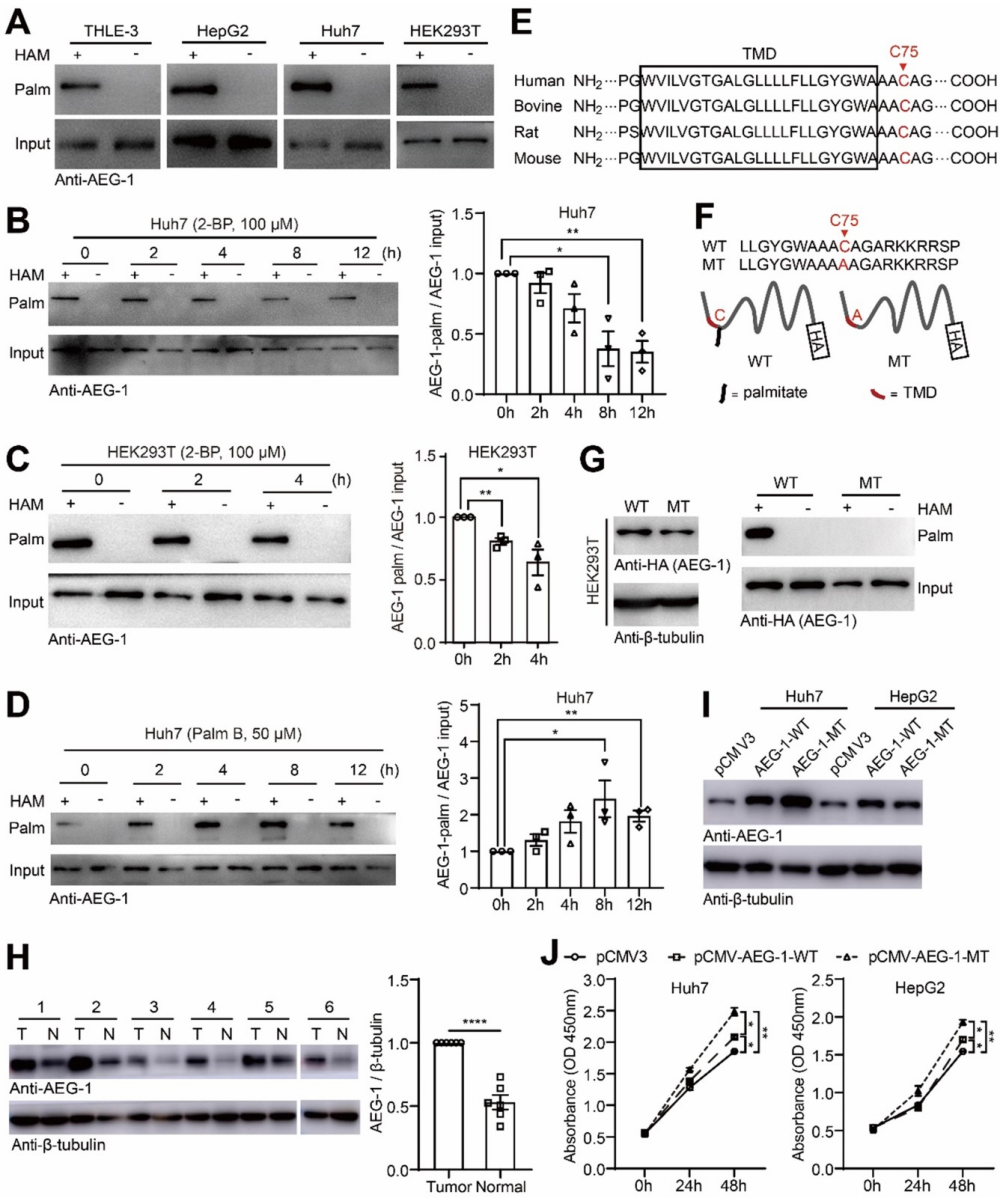
** AEG-1 is a palmitoylated protein and might be involved in regulating liver cancer cell proliferation. (A)** The palmitoylation of endogenous AEG-1 in THLE-3, HepG2, Huh7 and HEK293T cells was analyzed by Acyl-RAC assay. HAM+: with HAM, HAM-: without HAM. **(B, C)** Left, Huh7 and HEK293T cells were treated with 2-BP for different period of time, respectively, and then the level of AEG-1 palmitoylation was evaluated by Acyl-RAC assay. Right, quantification of the results. Data were normalized to 0 h. The experiment was repeated three times. **(D)** Left, Huh7 cells were treated with Palm B for different period of time, and then the level of AEG-1 palmitoylation was evaluated by Acyl-RAC assay. Right, quantification of the results. Data were normalized to 0 h. The experiment was repeated three times. **(E)** Cysteine conservation analysis of AEG-1 protein sequences in different species. **(F)** Schematic diagram of cysteine mutation at position 75 in AEG-1. **(G)** WT or MT AEG-1 were expressed in HEK-293T cells for detecting its palmitoylation level by Acyl-RAC assay. The experiment was repeated three times. **(H)** The level of AEG-1 expression in tumor tissues and adjacent normal tissues were detected by western blotting. T: tumor tissue, N: normal tissue. Data were normalized to tumor tissue. β-tubulin was used as a loading control. **(I)** AEG-1-WT and AEG-1-MT were overexpressed in Huh7 and HepG2 cells, respectively. β-tubulin was used as a loading control, and the experiment was repeated three times. **(J)** The CCK-8 assay was applied to measure cell proliferation in Huh7 and HepG2 cells expressing either pCMV3 empty vector, pCMV-AEG-1-WT or pCMV-AEG-1-MT plasmid (n=3). The data are presented as the mean ± SEM. *p < 0.05, **p < 0.01, ****p < 0.0001. Statistical significance was determined by unpaired two-tailed Student's t-test for comparison between two groups and one-way ANOVA with Dunnett's multiple comparison test for comparison among three groups.

Repeatedly, AEG-1 was suggested to be potentially palmitoylated in numerous palmitoyl-protein proteomics (http://swisspalm.epfl.ch/), which however has not been verified. To this end, we examined AEG-1 palmitoylation in several cell lines and the results showed that AEG-1 is readily palmitoylated in THLE-3, HepG2, Huh7 and HEK293T cells (Figure 1A). For prove in principal, Huh7 and HEK293T cells were treated with 2-BP, a general palmitoylation inhibitor 31, and it was found that 2-BP gradually suppressed the palmitoylation level of AEG-1 in a time-dependent manner (Figure 1B-C); in contrast, Palm B, a general depalmitoylation inhibitor, significantly enhanced the level of AEG-1 palmitoylation in Huh7 cells (Figure 1D), suggesting that AEG-1 palmitoylation can be dynamically regulated. Moreover, to map the specific palmitoylation site, AEG-1 protein sequences from multiple species were aligned and it showed that cysteine at position 75 (Cys-75) is well conserved across species, also featured by a close location to the transmembrane domain at the N terminus (Figure 1E). Interestingly, Cys-75 is the only residue available for palmitoylation in human AEG-1 protein. Accordingly, Cys-75 was mutated into alanine (AEG-1-MT) in AEG-1 (Figure 1F) and surprisingly, such mutation completely abolished palmitoylation in AEG-1-MT (Figure 1G), proving that Cys-75 is the specific site of palmitoylation in AEG-1.

As previously reported that the protein level of AEG-1 is often found upregulated in different type of tumors 32, 33, consistently, here we showed that AEG-1 expression is also augmented in HCC tumor tissues as compared to their adjacent normal tissues (Figure 1H), indicating that increased levels of AEG-1 might promote HCC tumorigenesis. To explore whether AEG-1 palmitoylation is linked to HCC tumorigenesis, AEG-1-WT or AEG-1-MT was expressed in Huh7 and HepG2 cells (Figure 1I), and the results showed that the expression of AEG-1-MT boosted cell growth as compared to the control (transfected with empty vector), while the expression of AEG-1-MT further accelerated cell proliferation as compared to that of the WT AEG-1 (Figure 1J). Together, these results implied that palmitoylation might be a functional switch for AEG-1 to regulate liver cancer cell proliferation.

### Blocking AEG-1 palmitoylation *in vivo* exacerbates the progression of DEN-induced HCC

**Figure 2:**
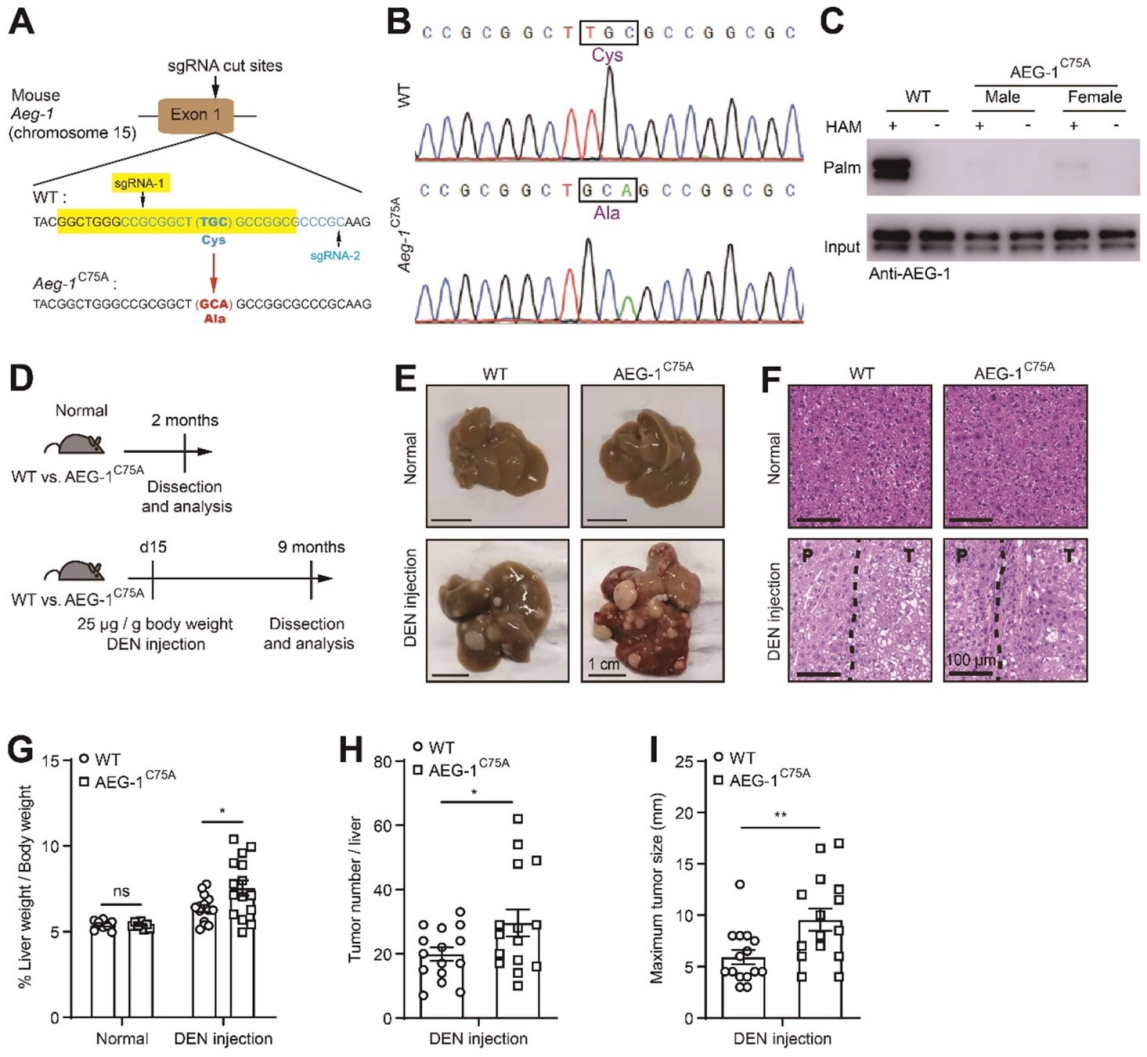
** Blocking AEG-1 palmitoylation *in vivo* exacerbates the progression of DEN-induced HCC. (A)** Schematic diagram of the point mutation (C75A) in exon 1 of mouse AEG-1.** (B)** Sequencing result of WT and AEG-1^C75A^ mice. The mutation of TGC to GCA was boxed and the translated amino acid was labeled in purple.** (C)** Palmitoylation levels of AEG-1 in livers of WT and AEG-1^C75A^ mice were detected by Acyl-RAC assay. The experiment was repeated three times.** (D)** Schematic diagram of DEN-induced HCC model. **(E, F)** Representative images of whole-liver morphology (**E**) and H&E staining (**F**) of liver sections from normal and DEN-treated WT and AEG-1^C75A^ mice. P: paratumor tissue; T: tumor tissue. **(G-I)** Quantifications of liver weight/body weight ratio, tumor number, and maximum tumor size (n=8 of normal mice, n=15 of DEN-treated mice). The data are presented as the mean ± SEM. *p < 0.05, **p < 0.01, ns: no significance. Statistical significance was determined by unpaired two-tailed Student's t-test for comparison between two groups.

To further verify the roles of AEG-1 palmitoylation in HCC progression *in vivo*, a knock-in mice was generated with the ablation of AEG-1 palmitoylation (C75A, AEG-1^C75A^) in C57BL/6 background (Figure 2A-C). To assess the possible impact on the immune system caused by AEG-1 mutation, we analyzed the single-cell sequencing data from human liver hepatocellular carcinoma (LIHC) samples from the TISCH website 34, and found that AEG-1 is highly expressed in a variety of immune cells (Figure S1A). Therefore, flow cytometry analysis was performed and the corresponding results showed that the absolute number of white blood cells (WBC), T cells, B cells, neutrophils, CD4^+^ T cells, CD8^+^ T cells and macrophages in mouse liver were not apparently altered in AEG-1^C75A^ as compared to WT mice (Figure S1B-G). Next, we intended to explore if the ablation of AEG-1 palmitoylation (C75A) might contribute to HCC tumorigenesis by incorporating a HCC model induced by intraperitoneal injection of DEN 35 (Figure 2D). Under normal chow diet, both AEG-1^C75A^ and WT mice exhibited no spontaneous liver injury or malignancies at the age of 2 months, whereas DEN-treated WT and AEG-1^C75A^ mice developed typical HCC accompanied with fibrosis, as presented by the whole liver morphology (Figure 2E) and H&E analysis (Figure 2F) at the age of 9 months. Notably, the liver weight/body weight ratio, tumor number, and maximum tumor diameter were all significantly increased in AEG-1^C75A^ mice as compared with WT mice (Figure 2G-I). Together, these data clarified the notion that loss of AEG-1 palmitoylation might accelerates HCC progression in DEN-induced HCC mouse model.

### AEG-1 is palmitoylated by zDHHC6 and the latter correlates positively with patient survival in HCC

**Figure 3:**
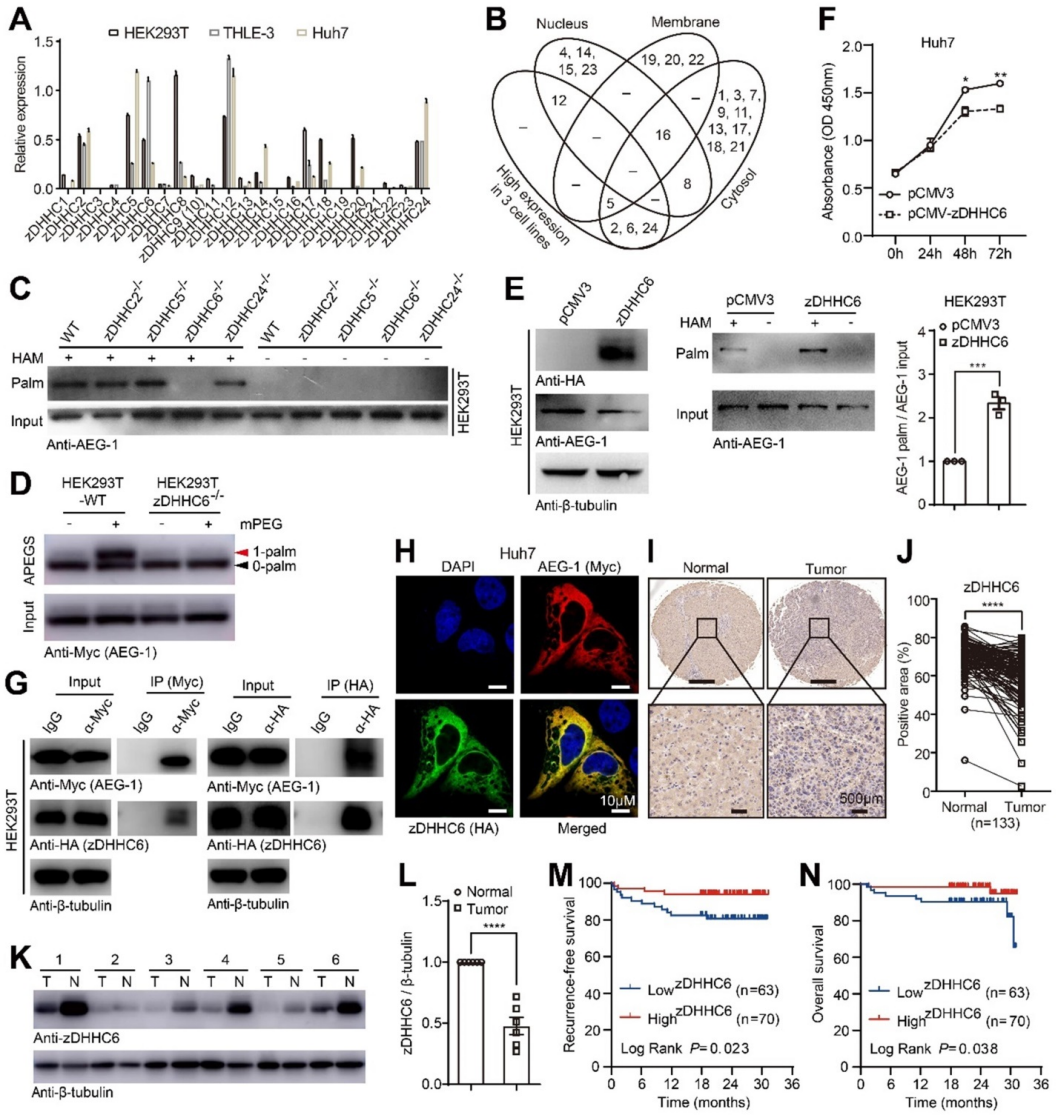
**AEG-1 is palmitoylated by zDHHC6 and the latter correlates positively with patient survival in HCC. (A)** The relative expression profile of zDHHCs was measured by qRT-PCR in varied cell lines (n=3). **(B)** Venn diagram showing the expression level and subcellular distribution of zDHHCs in HCC. **(C)** The palmitoylation level of endogenous AEG-1 was measured in either WT or genetically modified HEK293T cells. The experiment was repeated three times.** (D)** The APEGS assay was performed to detect the palmitoylation level of exogenous AEG-1 expressed in WT or zDHHC6^-/-^ HEK293T cells. 0-palm: no palmitoylation; 1-palm: one palmitoylation. The experiment was repeated three times.** (E)** HEK293T cells expressing either pCMV3 or HA-tagged zDHHC6 were examined for the level of AEG-1 palmitoylation (left), and quantified (right). Data were normalized to pCMV3. The experiment was repeated three times.** (F)** The CCK-8 assay was used to measure cell proliferation in Huh7 cells expressing either pCMV3 or pCMV-zDHHC6 plasmid (n=3).** (G)** Myc-tagged AEG-1 and HA-tagged zDHHC6 were co-expressed in HEK293T cells, and the association of AEG-1 with zDHHC6 was detected by co-IP and western blotting. The experiment was repeated three times.** (H)** AEG-1 (Myc) and zDHHC6 (HA) were immunostained in Huh7 cells, Scale bar, 10 μm. **(I, J)** Protein level of zDHHC6 was examined in HCC and the adjacent normal tissues by IHC, and quantified (n=133).** (K, L)** Western blot analysis of zDHHC6 expression in HCC and the adjacent normal tissues, and quantified (n=6). T: tumor, N: normal. Data were normalized to tumor tissue. **(M, N)** Kaplan-Meier analysis of recurrence-free survival and overall survival in HCC patients after stratifying them by the defined zDHHC6 IHC score. The data are presented as the mean ± SEM. *p < 0.05, **p < 0.01, ***p < 0.001, ****p < 0.0001. Statistical significance was determined by unpaired two-tailed Student's t-test for comparison between two groups.

As shown earlier that AEG-1 palmitoylation is dynamic (Figure 1B-D), it is timing to understand which enzyme catalyzed AEG-1 palmitoylation. To such end, we established a data repertoire comprising of the mRNA expression of all zDHHCs, subcellular localization and loss-of-function approaches. In light of the facts that AEG-1 is mainly localized in the cytoplasm 36, 37, which is also palmitoylated in all three human cell lines (HEK293T, THLE-3 and Huh7), we narrowed down the possibilities to enzymes that is mainly located within cytosol (The Human Protein Atlas) (Figure 3B), as well as possess an adequate amount of expression in all three cell lines (Figure 3A). Jointly, this raised zDHHC-2, -5, -6 and -24 as candidate palmitoyl-transferases for AEG-1. For verification, these zDHHCs were deleted in HEK293T cells via CRISPR/Cas9 (Figure S2), and the screening results showed that the knockout of zDHHC6, but not other zDHHCs, virtually abolished AEG-1 palmitoylation (Figure 3C). In addition, APEGS assay was used to confirm that WT AEG-1 is mono-palmitoylated, illustrated by a single band shift in APEGS (Figure 3D), whereas the depletion of zDHHC6 inhibits AEG-1 palmitoylation, indicated by no such band shift (Figure 3D). And reversibly, ectopically expressed zDHHC6 augmented the level of endogenous AEG-1 palmitoylation (Figure 3E), which inhibited the proliferation of Huh7 cells (Figure 3F). These data further strengthen the notion that AEG-1 palmitoylation negatively regulates the proliferation of liver cancer cells.

As enzyme should associate with its substrate, we went on to test if zDHHC6 binds to AEG-1. Indeed, the co-IP results demonstrated that zDHHC6 could pull down AEG-1 (Figure 3G). Meanwhile, ectopically expressed zDHHC6 colocalized nicely with AEG-1 in the cytosol of cultured Huh7 cells (Figure 3H).

To investigate the correlations between zDHHC6 and HCC tumorigenesis, a tissue microarray containing 133 pairs of HCC tumor and adjacent normal tissues was analyzed by IHC and the results showed that zDHHC6 expression (mainly within the cytoplasm) was significantly lower in HCC tumors than that of the adjacent normal tissues (Figure 3I-J). Moreover, we collected six pairs of clinical HCC tumor and adjacent normal tissues, and consistently, these results confirmed a decreased level of zDHHC6 in HCC samples (Figure 3K-L). Remarkably, lower level of zDHHC6 is significantly correlated with poor prognosis, illustrated by lowered recurrence-free survival (RFS, *P*=0.023) and overall survival (OS, *P*=0.038) (Figure 3M-N). Taken together, these data suggested that the zDHHC6-AEG-1 axis may play important roles in the progression of HCC.

### Eliminating Zdhhc6 in mice exacerbates the progression of DEN-induced HCC

**Figure 4:**
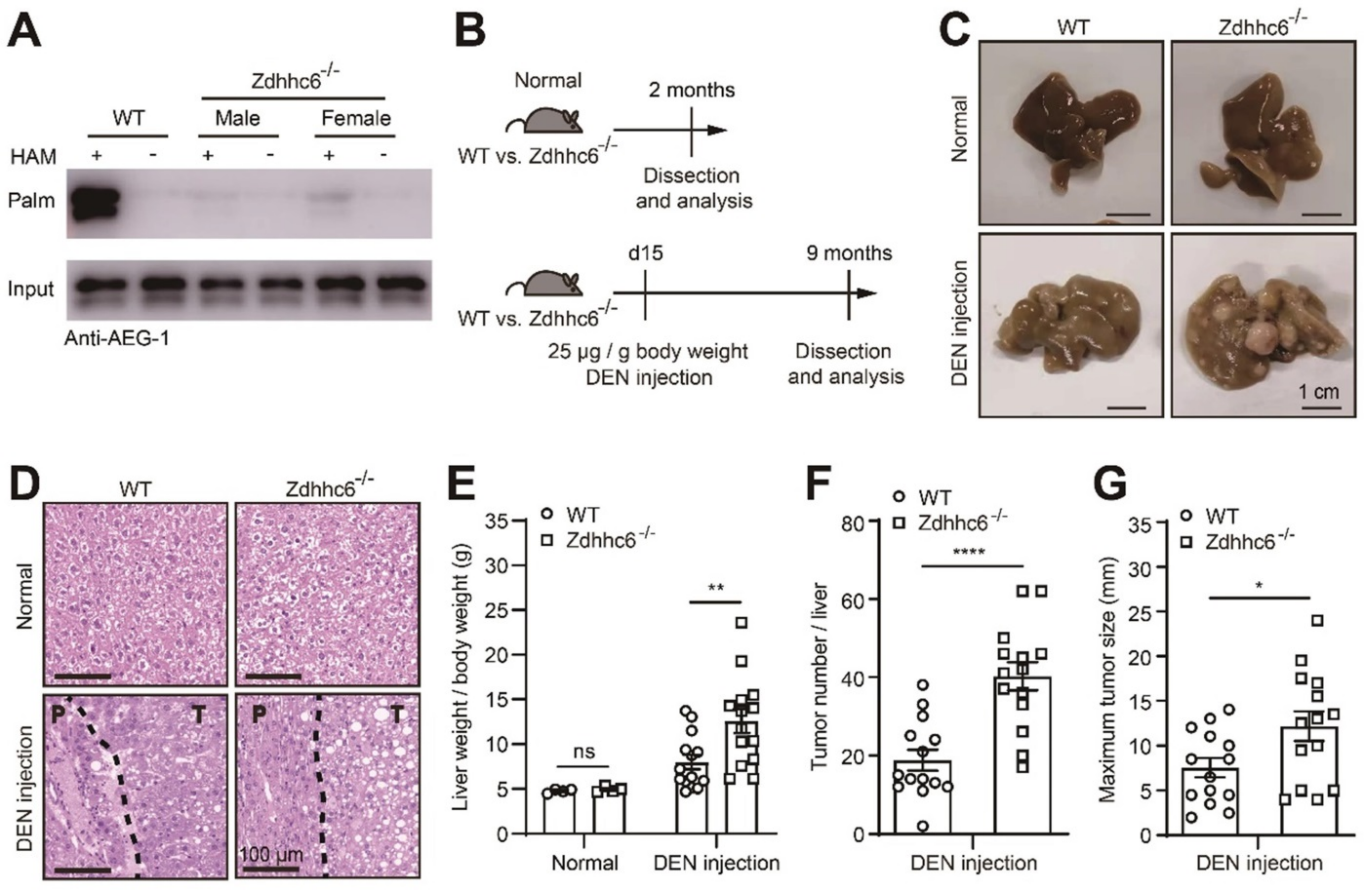
**Genetic deletion of Zdhhc6 exacerbates the progression of DEN-induced HCC. (A)** The level of AEG-1 palmitoylation was measured in livers of WT or Zdhhc6^-/-^ mice by Acyl-RAC assay. The experiment was repeated three times.** (B)** Schematic diagram of DEN-induced HCC model. **(C, D)** Representative images of whole-liver morphology and H&E staining of liver sections from normal or DEN-treated WT and Zdhhc6^-/-^ mice. P: paratumor tissue; T: tumor tissue. **(E-G)** The statistical analysis of liver weight/body weight ratio, tumor number, and maximum tumor size (n=4 of normal mice, n=14 of DEN-treated mice). The data are presented as the mean ± SEM. *p < 0.05, **p < 0.01, ****p < 0.0001, ns: no significance. Statistical significance was determined by unpaired two-tailed Student's t-test for comparison between two groups.

To further elucidate the roles of Zdhhc6 in HCC development, Zdhhc6 was depleted (Zdhhc6^-/-^) in mice using CRISPR/Cas9 (Figure S3), which also resulted in a nearly complete abolition of AEG-1 palmitoylation in mouse liver (Figure 4A). Likewise, to analyze the potential impact on the immune system upon Zdhhc6 removal, the single-cell sequencing data (from human LIHC samples from the TISCH website) pointed out that Zdhhc6 is expressed at very low levels in immune cells (Figure S4A) 34. As expected, the flow cytometry data showed that the knockout of Zdhhc6 does not affect the absolute number of WBC, T cells, B cells, neutrophils, CD4^+^ T cells, CD8^+^ T cells and macrophages in mouse liver (Figure S4B-E).

As shown earlier, DEN-induced HCC model was incorporated in Zdhhc6^-/-^ mouse (Figure 4B), similarly, all mice developed typical HCC accompanying with severe fibrosis (Figure 4C-D), and strikingly, parameters as the liver weight/body weight ratio, tumor number, and maximum tumor diameter were significantly elevated in Zdhhc6^-/-^ mice as compared with WT mice (Figure 4E-G), while no spontaneous liver injury or malignancies were observed in both genotypes on normal chow diet (Figure 4C-D). Echoing with the major conclusions in Figure 2, these combined results suggested the following logic that down-regulated AEG-1 palmitoylation, either caused by point mutation (C75A) or the deletion of Zdhhc6, strongly promotes HCC progression in DEN-induced mouse model.

### Loss of palmitoylation may inhibit proteasome-mediated degradation of AEG-1 and enhance AEG-1-SND1 interaction and RISC activity

**Figure 5:**
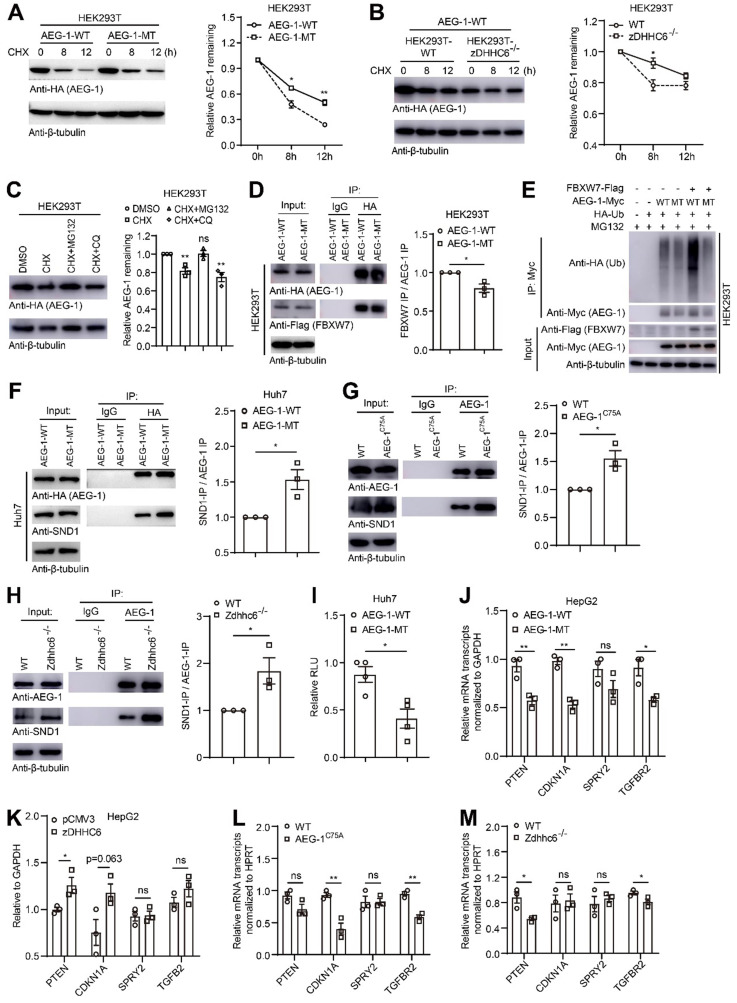
**Loss of palmitoylation may inhibit ubiquitination-mediated degradation of AEG-1 and enhance RISC activity via interacting with SND1. (A)** Left, HEK293T cells expressing AEG-1-WT or AEG-1-MT were treated with CHX and examined for the level of AEG-1. Right, quantification of the results. Data were normalized to 0 h. β-tubulin was used as a loading control, and the experiment was repeated three times.** (B)** Left, AEG-1-WT was expressed in WT or zDHHC6^-/-^ cells and treated with CHX for the measurement of AEG-1 protein level. Right, quantification of the results. Data were normalized to 0 h. β-tubulin was used as a loading control, and the experiment was repeated three times.** (C)** Left, HEK293T cells expressing AEG-1-WT were treated with either DMSO, CHX plus MG132 or CHX plus CQ for the measurement of AEG-1 protein level. Right, quantification of the results. Data were normalized to DMSO treatment. β-tubulin was used as a loading control, and the experiment was repeated three times.** (D)** Left, FBXW7 was co-expressed with either AEG-1-WT or AEG-1-MT for immunoprecipitation analysis, and quantified (right). Data were normalized to AEG-1-WT. The experiment was repeated three times.** (E)** Detecting AEG-1 ubiquitination in cells expressing HA-Ub, FBXW7-Flag, and AEG-1-Myc (WT or MT). The experiment was repeated three times. **(F)** AEG-1-WT or AEG-1-MT was expressed in Huh7 cells for the measurement of coprecipitation with SND1, and quantified. Data were normalized to AEG-1-WT. β-tubulin was used as a loading control, and the experiment was repeated three times.** (G)** Left, the association of AEG-1 with SND1 in livers of WT and AEG-1^C75A^ mice were analyzed by co-IP and western blotting, and quantified (right). Data were normalized to WT. β-tubulin was used as a loading control, and the experiment was repeated three times.** (H)** Left, the association of AEG-1 with SND1 in livers of WT and Zdhhc6^-/-^ mice were analyzed by co-IP and western blotting, and quantified (right). Data were normalized to WT. β-tubulin was used as a loading control, and the experiment was repeated three times.** (I)** RISC activity was measured in Huh7 cells. Rluc activity was normalized to Fluc activity (n=3). Data were normalized to AEG-1-WT.** (J, K)** Relative mRNA expression levels of several tumor suppressors (PTEN, CDKN1A, SPRY2 and TGFB2) was measured in conditioned HepG2 cells where either AEG-1-WT/-MT **(J)** or pCMV3/zDHHC6 **(K)** were expressed (n=3). Data were normalized to AEG-1-WT **(J)** or pCMV3 **(K)**.** (L)** mRNA expression levels of PTEN, CDKN1A, SPRY2 and TGFB2 in livers of WT and AEG-1^C75A^ mice (n=3). Data were normalized to WT. **(M)** mRNA expression levels of PTEN, CDKN1A, SPRY2 and TGFB2 in livers of WT and Zdhhc6^-/-^ mice (n=3). Data were normalized to WT. The data are presented as the mean ± SEM. *p < 0.05, **p < 0.01, ns: no significance. Statistical significance was determined by unpaired two-tailed Student's t-test for comparison between two groups.

It has been shown that palmitoylation plays vital roles in regulating protein stability and subcellular localization 14, 38. For such purpose, AEG-1-WT or AEG-1-MT was expressed and the fluorescent images showed that both proteins could localize on the plasma membrane and in the cytoplasm (Figure S5), implying that AEG-1 palmitoylation is irrelevant of its protein positioning. Next, we examined whether palmitoylation is relevant for AEG-1 stability. AEG-1-WT or AEG-1-MT was expressed and treated with DMSO or CHX (blocking *de novo* protein synthesis), and the results showed that AEG-1-MT exhibited a significantly lower rate of protein degradation than that of the WT AEG-1 (Figure 5A), supported also by the finding that the degradation rate of AEG-1-WT was inhibited upon the removal of zDHHC6 (Figure 5B). These results indicated that palmitoylation negatively regulates AEG-1 protein stability *ex vivo*.

To figure out the potential pathway that might degrade AEG-1, proteasomal inhibitor MG132 and lysosomal inhibitor CQ were applied and it showed that MG132 could drastically inhibit the degradation of AEG-1, suggesting that AEG-1 degradation is majorly mediated through the proteasome pathway (Figure 5C). As ubiquitinated proteins are mainly degraded by the proteasome pathway, and interestingly, it was reported that AEG-1 can be ubiquitinated by FBXW7 (an E3 ubiquitin ligase) in breast cancer cells 7. Therefore, we examined the interaction between AEG-1 and FBXW7, and the IP experiments presented that AEG-1-MT appears to have a weak binding with FBXW7 as compared to WT AEG-1 (Figure 5D), which possibly leads to reduced level of ubiquitination and enhanced protein stability in AEG-1-MT vs AEG-1-WT (Figure 5E).

Furthermore, it was demonstrated that AEG-1 interacts with SND1 to be part of the RNA-induced silencing complex (RISC), and enhanced RISC activity may inhibit the expression of tumor suppressor genes 6. Notably, it was found that reduced level of AEG-1 palmitoylation, either by C75A mutation or the deletion of zDHHC6, rendered a higher affinity with SND1 as compared to WT AEG-1 (Figure 5F-H). As a consequence, the RISC activity is significantly upregulated as shown by the diminish of RLU value (Figure 5I), as well as its downstream targets (PTEN, CDKN1A, SPRY2 and TGFBR2) in cells expressing AEG-1-MT vs AEG-1-WT (Figure 5J). And vice versa, increasing AEG-1 palmitoylation by zDHHC6 expression upregulated the expression level of tumor suppressor genes (Figure 5K). Additional evidences also came from the mouse model that AEG-1 palmitoylation positively correlated with the expression of tumor suppressor genes (Figure 5L and Figure 5M).

In total, these data may reveal a pathological mechanism that reduced level of AEG-1 palmitoylation stimulates HCC progression though controlling its protein stability as well as regulating RISC activity to downregulate tumor suppressors.

### AEG-1 is likely depalmitoylated by PPT1 and PPT2

**Figure 6:**
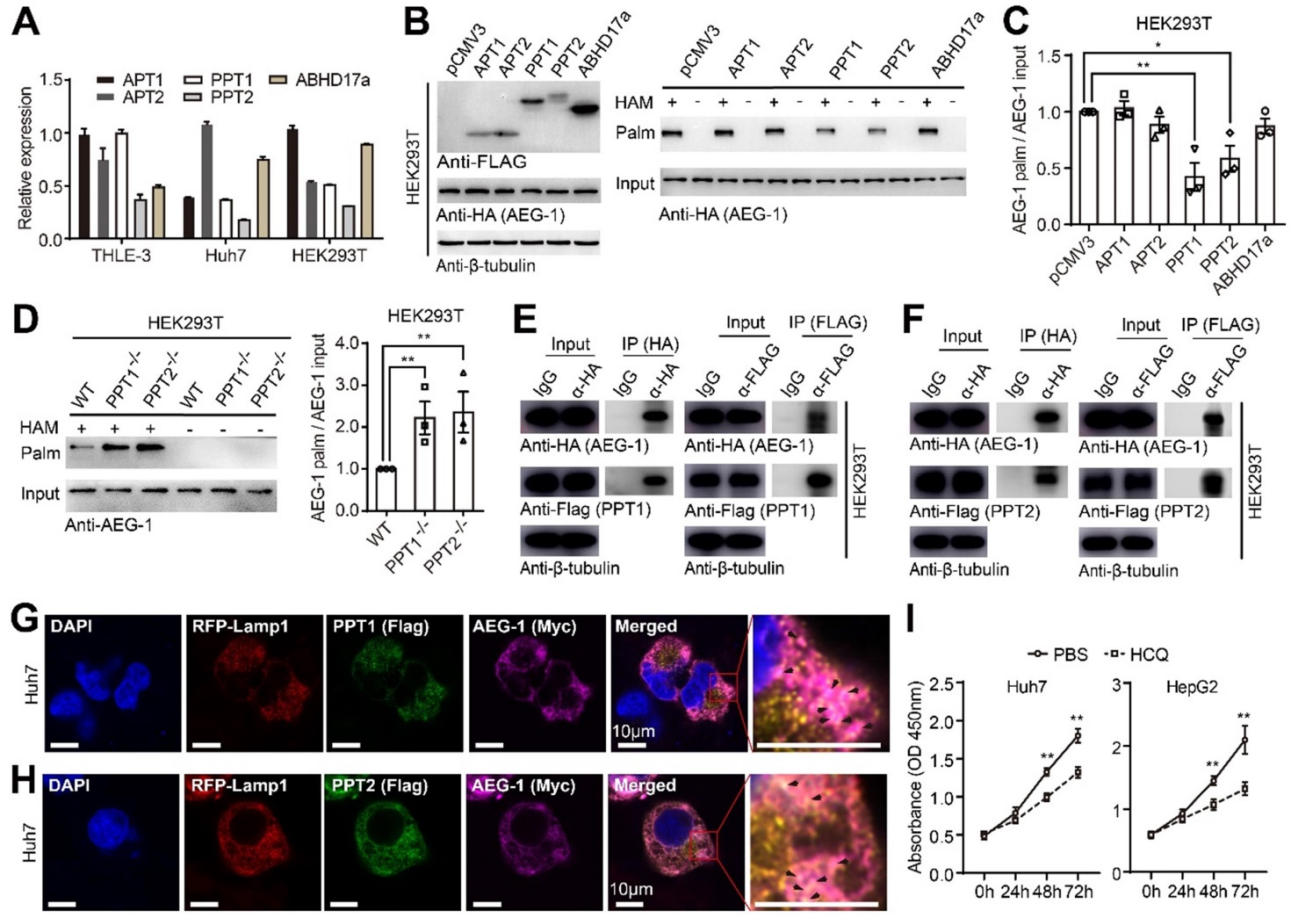
**AEG-1 is potentially depalmitoylated by PPT1 and PPT2. (A)** The mRNA expression profiles of different PPTs were examined in varied cell lines (n=3).** (B, C)** AEG-1-WT was co-expressed with PPTs in HEK293T cells and measured for the level of AEG-1 palmitoylation** (B)**, which was quantified **(C)**. Data were normalized to pCMV3. β-tubulin was used as a loading control, and the experiment was repeated three times.** (D)** Left, the palmitoylation level of AEG-1 were analyzed in WT, PPT1^-/-^ and PPT2^-/-^ HEK293T cells by Acyl-RAC assay, and quantified (right). The experiment was repeated three times.** (E, F)** The interaction of AEG-1 with PPT1** (E)** or PPT2** (F)** was detected by co-IP and western blotting. β-tubulin was used as a loading control, and the experiment was repeated three times.** (G, H)** Huh7 cells were immunostained for AEG-1 (Myc) and PPT1 (FLAG, **G**) or PPT2 (FLAG,** H**), Scale bar, 10 μm. **(I)** The CCK-8 assay was used to measure cell proliferation in Huh7 and HepG2 cells treated with 1x PBS or HCQ (n=3). The data are presented as the mean ± SEM. *p < 0.05, **p < 0.01. Statistical significance was determined by unpaired two-tailed Student's t-test for comparison between two groups.

To identify the enzymes catalyzing the depalmitoylation of AEG-1, the expression patterns of the most common thioesterases including APT1/2, PPT1/2 and ABHD17a were profiled and it showed that all these genes were well expressed in different cell lines (Figure 6A). Additionally, they were individually co-expressed with HA-tagged AEG-1 for the evaluation of AEG-1 palmitoylation, and the results illustrated that PPT1 or PPT2 could significantly lower the palmitoylation of AEG-1 (Figure 6B-C). For confirmation, PPT1 or PPT2 was deleted in HEK293T cells (Figure S6A-D) and the corresponding experiments demonstrated that the level of AEG-1 palmitoylation is greatly elevated in PPT1^-/-^ or PPT2^-/-^ cells (Figure 6D). Moreover, the co-IP experiments indicated that PPT1 and PPT2 are able to bind to AEG-1 (Figure 6E-F), supported also by the immunofluorescence staining that PPT1 or PPT2 partial colocalizes with AEG-1 in Huh7 cells (Figure 6G-H).

As shown earlier that non-palmitoylated AEG-1 accelerates cell proliferation (Figure 1J), we tested the hypothesis if enhanced level of AEG-1 palmitoylation might decelerate cell growth by targeting PPT1. Interestingly, HCQ was introduced by other study to inhibit PPT1 activity 24. Therefore, HCQ was used to incubate with Huh7 and HepG2 cells and the CCK-8 assays showed that HCQ could dramatically slow down the proliferation of these cells (Figure 6I), suggesting of potential pharmaceutical values of targeting AEG-1 palmitoylation by HCQ.

### Enhancing AEG-1 palmitoylation by HCQ suppresses tumor growth of HCC in xenograft model

**Figure 7:**
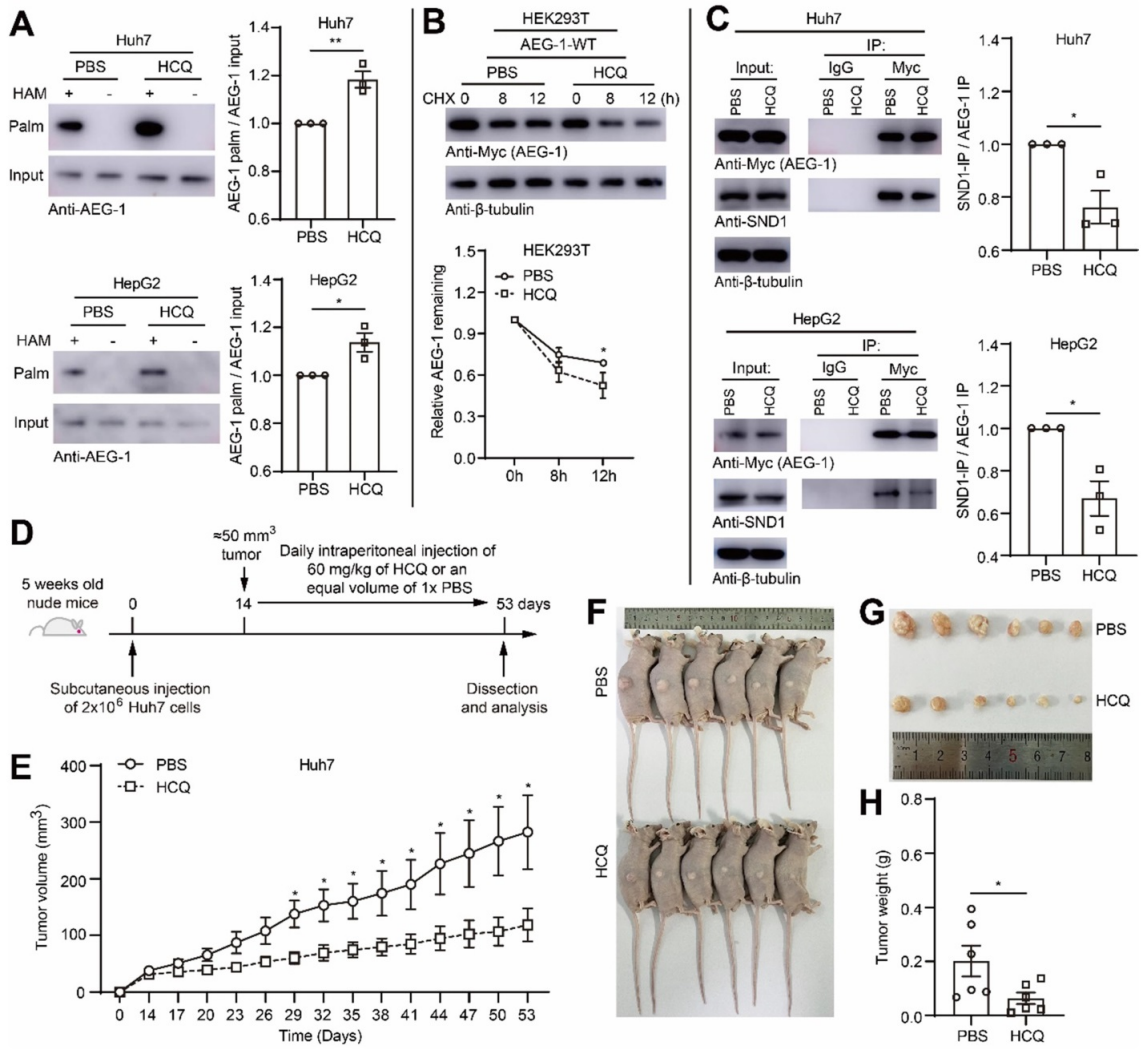
**Enhancing AEG-1 palmitoylation by HCQ suppresses tumor growth of HCC in xenograft models. (A)** Huh7 and HepG2 cells were treated with 1× PBS or HCQ for the measurement of AEG-1 palmitoylation. Data were normalized to PBS treatment. The experiment was repeated three times.** (B)** Left, HEK293T cells expressing AEG-1-WT were treated with 1× PBS or HCQ for the examination of protein stability in the presence of CHX, and quantified (right). Data were normalized to 0 h. β-tubulin was used as a loading control, and the experiment was repeated three times.** (C)** The interaction of AEG-1 with SND1 was analyzed by co-IP in Huh7 or HepG2 cells expressing AEG-1-Myc while treated with 1× PBS or HCQ. Data were normalized to PBS treatment. β-tubulin was used as a loading control, and the experiment was repeated three times.** (D)** Schematic diagram of subcutaneous tumor formation and intraperitoneal injection of 1× PBS or HCQ in nude mice.** (E-H)** Huh7 cells were subcutaneous transplanted into the right flank of Bal/bc nude mice (6 mice in each group). Tumor volume was measured every 3 days after intraperitoneal injection of 1× PBS or HCQ. Mice were sacrificed 53 days after implantation, and tumors were isolated, photographed and weighed. The data are presented as the mean ± SEM. *p < 0.05, **p < 0.01. Statistical significance was determined by unpaired two-tailed Student's t-test for comparison between two groups.

To test the idea whether HCQ directed PPT1 inhibition might bring pharmaceutical benefits for HCC progression, we applied HCQ in mouse xenograft model. However, initially, it was ensured that HCQ indeed substantially elevated the level of AEG-1 palmitoylation in Huh7 and HepG2 cells (Figure 7A), which subsequently also promoted AEG-1 degradation (Figure 7B) and inhibited AEG-1 and SND1 interaction (Figure 7C). Later on, Huh7 cells was inoculated into nude mice by subcutaneous injection, HCQ or 1 × PBS was used for treatment by intraperitoneal injection (Figure 7D). The results of the xenograft model showed that the intervention of HCQ indeed rapidly reduced tumor growth featured by diminished tumor volume and tumor weight as compared to that of the control group (Figure 7E-H). Although briefly, these experiments did suggest that AEG-1 palmitoylation might be a valuable pharmaceutical target and further, increasing AEG-1 palmitoylation by HCQ might confer therapeutic benefits for HCC patients in clinics, which warrants future investigations.

## Discussion

**Figure 8:**
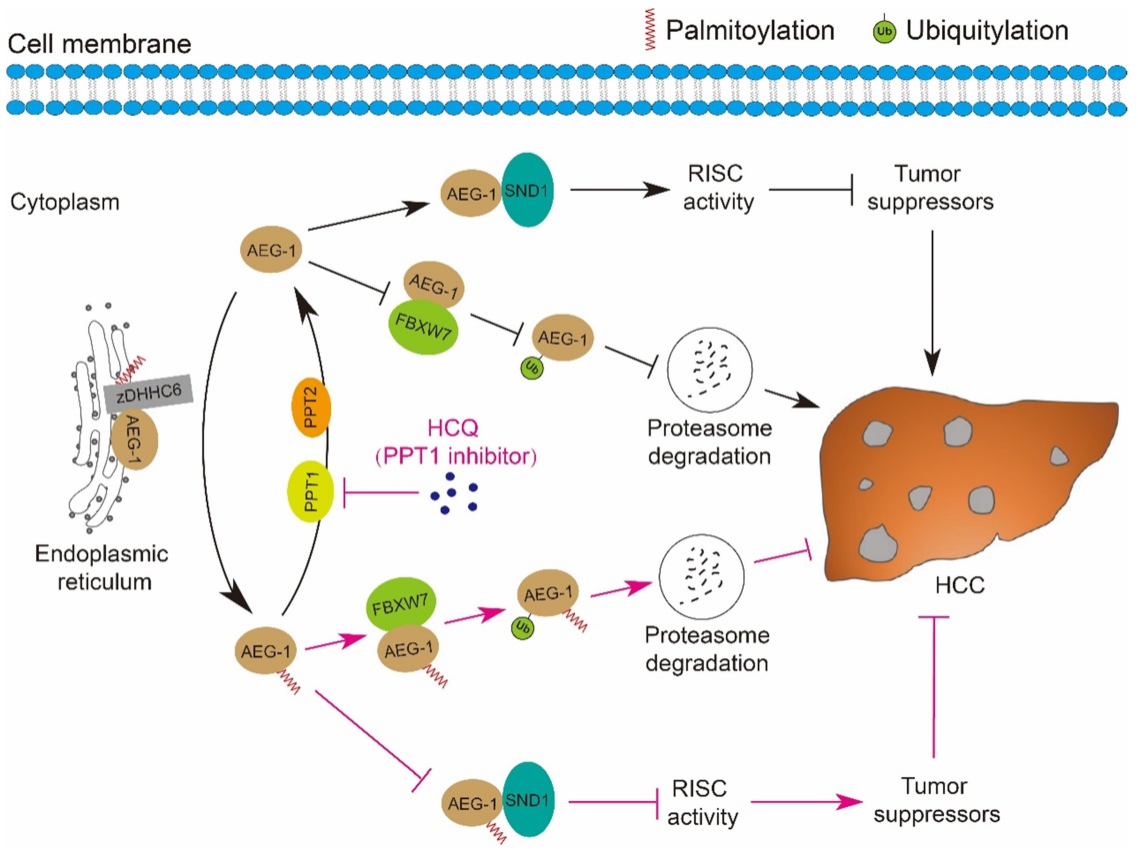
**A schematic diagram illustrates the mechanisms by which the palmitoylation of AEG-1 regulates the progression of HCC.** zDHHC6 palmitoylates AEG-1 to promote its interaction with the E3 ubiquitin ligase FBXW7, which then enhances the ubiquitination level and proteasome-mediated degradation of AEG-1, ultimately suppressing HCC progression. Reversibly, PPT1 and PPT2 depalmitoylate AEG-1 and increase the interaction of AEG-1 with SND1, thereby enhancing RISC activity and possibly inhibiting the expression of tumor suppressors, eventually facilitating the progression of HCC. Intriguingly, inhibition of PPT1 activity by HCQ promotes the degradation of AEG-1 and weakens the interaction between AEG-1 and SND1, thereby suppressing the growth of HCC.

Fast expanding in the research field of understanding the roles of protein palmitoylation in different types of human malignancies has been achieved in the past decades 39-41, however, it was revealed that the linkage between palmitoylation and HCC is relatively weak as compared to other tumor types, e.g. breast cancer, prostate cancer, colorectal cancer, leukemia, melanoma, pancreatic cancer and NSCLC 20, 23, 42-48. Our current study strengthened the connection that AEG-1, a key driver of HCC, is palmitoylated, which negatively regulates its protein stability via proteasome mediated degradation pathway; inhibiting AEG-1 palmitoylation enhances its binding to SND1 and thus the RISC activity, the latter may downregulate the mRNA level of several tumor suppressors as PTEN/TGFB2 and therefore promotes HCC progression. Notably, such palmitoylation is dynamic, as AEG-1 palmitoylation is reversibly manipulated by zDHHC6 and PPT1/2 in both directions. And importantly, targeting PPT1 by HCQ to augment the palmitoylation level of AEG-1 successfully suppressed liver cancer cell growth (Figure 8).

AEG-1 is a multifaceted protein involved in not only growth and proliferation, angiogenesis, metastasis and chemoresistance, engaging very complicated signaling cascades, for example, AEG-1 mediated PI3K/AKT induction activates a series of phosphorylation processes, which promotes c-MYC activity, inactivates GSK3β and more production of AEG-1, while inhibits the activity and the production of p53, may accompanied by suppressing the starvation-dependent p21/mda-6 expression and activating MDM2, with the consequence of stimulating tumor progression 49. Moreover, SND1 was suggested to interact with AEG-1 in the cytoplasm to activate the RISC activity, the latter further controlled the mRNA expression of a list of tumor suppressors as PTEN, CDKN1A (p21), SPRY2, and TGFBR2 6. In agreement, such scenario was supported by our observations that stabilized AEG-1 (AEG-1^C75A^) enhances its binding to SND1 and activates RISC activity for the downregulation of these tumor suppressors and HCC progression (Figure 2, Figure 4 and Figure 5). To facilitate tumor invasion, AEG-1 was indicated to enhance the expression of a group of metalloproteinase e.g. MMP-2 and MMP-9 to degrade extracellular matrix for path clearing 50. Whether AEG-1 palmitoylation is also involved in regulating MMPs or PI3K/AKT signaling cascades was not investigated here and warrants further studies.

It was shown that AEG-1 binds MDM2 via its N-terminal domain to prevent the degradation of MDM2 through the ubiquitination-proteasome pathway 51. And similarly, AEG-1 inhibits the proteasomal degradation of FOXM1, leading to FOXM1 stabilization and overexpression of downstream targets 52. Interestingly, here we presented that the degradation of AEG-1 itself is also mediated by the ubiquitination-proteasome pathway, suggesting a notion that AEG-1 and its binding partners might be degraded by the same mechanistical machineries. Indeed, AEG-1 was demonstrated to interact with FBXW7 for ubiquitination in both breast cancer cells 7 and liver cancer cells (Figure 5D), implying a conserved protein degradation pathway related to AEG-1. More interestingly, the loss of AEG-1 palmitoylation confers more stability in itself by manipulating its binding affinity with FBXW7, extending the evidences that protein ubiquitination is frequently cross-talking with other form of PTMs 8, 9, in particular palmitoylation 8, 53-56.

As AEG-1 palmitoylation is dynamic (Figure 1), here we showed that such reversible process is catalyzed by zDHHC6 (Figure 3) and PPT1/2 (Figure 6). Although unknown, it can be assumed that the substrates of zDHHC6 are not only AEG-1 in HCC, truly, previous studies did identify several substrates of zDHHC6, e.g. CANX on mitochondria 57, NRAS, gp78 and IP3R on endoplasmic reticulum in different circumstances 58-60. In particular, targeting NRAS palmitoylation by artemisinin (a clinically approved antimalarial endoperoxide natural product) attenuates the ERK and AKT phosphorylation 60. Undoubtedly, the existence of multiple substrates of zDHHC6 does add extra complexity in interpreting the data presented here. However, considering the facts that the deletion of Zdhhc6 (Figure 4) virtually phenocopies that of the AEG-1^C75A^ (Figure 2) in the DEN-induced HCC mouse model, and AEG-1 is a proven substrate of zDHHC6 (Figure 3A-F), we tend to conceive that the zDHHC6-AEG-1 axis plays vital roles in regulating HCC progression, although we should not rule out other possibilities.

The most prominent feature of AEG-1 is that it is highly overexpressed in almost all types of tumors, making the knockdown of AEG-1 a preferred strategy for targeting tumor progression. Successful experience has been gained by the means of small interfering ribonucleic acid (siRNA) 61 and peptidomimetic inhibitors 62, and further, alternative inhibitory strategies are anticipated based on the unraveling of novel mechanisms. Coincidently, our data here suggested a mechanism that AEG-1 stability is negatively controlled by its site-specific palmitoylation, and increasing AEG-1 palmitoylation via inhibiting PPT1 by HCQ restrains HCC progression. These findings not only highlight the importance of dynamic AEG-1 palmitoylation in HCC, but also prove in principal that targeting AEG-1 palmitoylation might be a promising therapeutic strategy for treating HCC in clinics.

## Supplementary Material

Supplementary figures and tables.

## Abbreviations

AEG-1: Astrocyte-elevated gene-1
SND1: staphylococcal nuclease and tudor domain containing 1
HCC: hepatocellular carcinoma
RISC: RNA-induced silencing complex
PTM: post-translational modifications

## References

[1] Kochanek DM, Wells DG (2013). CPEB1 regulates the expression of MTDH/AEG-1 and glioblastoma cell migration. Mol Cancer Res.

[2] Huang S, Wu B, Li D, Zhou W, Deng G, Zhang K (2014). Knockdown of astrocyte elevated gene-1 inhibits tumor growth and modifies microRNAs expression profiles in human colorectal cancer cells. Biochemical and biophysical research communications.

[3] Sarkar D (2013). AEG-1/MTDH/LYRIC in liver cancer. Adv Cancer Res.

[4] Yao Y, Gu X, Liu H, Wu G, Yuan D, Yang X (2014). Metadherin regulates proliferation and metastasis via actin cytoskeletal remodelling in non-small cell lung cancer. British journal of cancer.

[5] Xu C, Kong X, Wang H, Zhang N, Kong X, Ding X (2014). MTDH mediates estrogen-independent growth and tamoxifen resistance by down-regulating PTEN in MCF-7 breast cancer cells. Cellular physiology and biochemistry: international journal of experimental cellular physiology, biochemistry, and pharmacology.

[6] Yoo BK, Santhekadur PK, Gredler R, Chen D, Emdad L, Bhutia S (2011). Increased RNA-induced silencing complex (RISC) activity contributes to hepatocellular carcinoma. Hepatology.

[7] Chen X, Li XY, Long M, Wang X, Gao ZW, Cui Y (2018). The FBXW7 tumor suppressor inhibits breast cancer proliferation and promotes apoptosis by targeting MTDH for degradation. Neoplasma.

[8] Yao H, Lan J, Li CS, Shi HB, Brosseau JP, Wang HB (2019). Inhibiting PD-L1 palmitoylation enhances T-cell immune responses against tumours. Nat Biomed Eng.

[9] Swaney DL, Beltrao P, Starita L, Guo A, Rush J, Fields S (2013). Global analysis of phosphorylation and ubiquitylation cross-talk in protein degradation. Nat Methods.

[10] Peng T, Hang HC (2015). Bifunctional fatty acid chemical reporter for analyzing S-palmitoylated membrane protein-protein interactions in mammalian cells. J Am Chem Soc.

[11] Ziemlińska E, Sobocińska J, Świątkowska A, Hromada-Judycka A, Traczyk G, Malinowska A (2021). Palm oil-rich diet affects murine liver proteome and S-palmitoylome. Int J Mol Sci.

[12] Pradhan AJ, Lu D, Parisi LR, Shen S, Berhane IA, Galster SL (2021). Protein acylation by saturated very long chain fatty acids and endocytosis are involved in necroptosis. Cell Chem Biol.

[13] Aramsangtienchai P, Spiegelman NA, Cao J, Lin H (2017). S-palmitoylation of junctional adhesion molecule C regulates its tight junction localization and cell migration. J Biol Chem.

[14] Kaur I, Yarov-Yarovoy V, Kirk LM, Plambeck KE, Barragan EV, Ontiveros ES (2016). Activity-dependent palmitoylation controls synDIG1 stability, localization, and function. J Neurosci.

[15] Zhu YC, Li D, Wang L, Lu B, Zheng J, Zhao SL (2013). Palmitoylation-dependent CDKL5-PSD-95 interaction regulates synaptic targeting of CDKL5 and dendritic spine development. Proc Natl Acad Sci USA.

[16] Holland SM, Collura KM, Ketschek A, Noma K, Ferguson TA, Jin Y (2016). Palmitoylation controls DLK localization, interactions and activity to ensure effective axonal injury signaling. Proc Natl Acad Sci USA.

[17] Das AK, Bellizzi JJ 3rd, Tandel S, Biehl E, Clardy J, Hofmann SL (2000). Structural basis for the insensitivity of a serine enzyme (palmitoyl-protein thioesterase) to phenylmethylsulfonyl fluoride. J Biol Chem.

[18] Duncan JA, Gilman AG (2002). Characterization of Saccharomyces cerevisiae acyl-protein thioesterase 1, the enzyme responsible for G protein alpha subunit deacylation *in vivo*. J Biol Chem.

[19] Lin DT, Conibear E (2015). ABHD17 proteins are novel protein depalmitoylases that regulate N-Ras palmitate turnover and subcellular localization. Elife.

[20] Chen S, Zhu B, Yin C, Liu W, Han C, Chen B (2017). Palmitoylation-dependent activation of MC1R prevents melanomagenesis. Nature.

[21] Zhang ZX, Li X, Yang F, Chen C, Liu P, Ren Y (2021). DHHC9-mediated GLUT1 S-palmitoylation promotes glioblastoma glycolysis and tumorigenesis. Nature communications.

[22] Pei X, Li KY, Shen Y, Li JT, Lei MZ, Fang CY (2022). Palmitoylation of MDH2 by ZDHHC18 activates mitochondrial respiration and accelerates ovarian cancer growth. Sci China Life Sci.

[23] Zhou B, Hao Q, Liang Y, Kong E (2022). Protein palmitoylation in cancer: molecular functions and therapeutic potential. Molecular oncology.

[24] Rebecca VW, Nicastri MC, Fennelly C, Chude CI, Barber-Rotenberg JS, Ronghe A (2019). PPT1 promotes tumor growth and is the molecular target of chloroquine derivatives in cancer. Cancer Discov.

[25] Xiong X, Hao Y, Sun K, Li J, Li X, Mishra B (2012). The Highwire ubiquitin ligase promotes axonal degeneration by tuning levels of Nmnat protein. PLoS Biol.

[26] Yuan W, Lu LX, Rao MD, Huang Y, Liu CE, Liu S (2021). GFAP hyperpalmitoylation exacerbates astrogliosis and neurodegenerative pathology in PPT1-deficient mice. Proc Natl Acad Sci USA.

[27] Wang X, Huang R, Zhang L, Li S, Luo J, Gu Y (2018). A severe atherosclerosis mouse model on the resistant NOD background. Dis Model Mech.

[28] Yokoi N, Fukata Y, Sekiya A, Murakami T, Kobayashi K, Fukata M (2016). Identification of PSD-95 Depalmitoylating Enzymes. J Neurosci.

[29] Zhang W, Zhangyuan G, Wang F, Jin K, Shen H, Zhang L (2021). The zinc finger protein Miz1 suppresses liver tumorigenesis by restricting hepatocyte-driven macrophage activation and inflammation. Immunity.

[30] Lochab S, Pal P, Kapoor I, Kanaujiya JK, Sanyal S, Behre G (2013). E3 ubiquitin ligase Fbw7 negatively regulates granulocytic differentiation by targeting G-CSFR for degradation. Biochim Biophys Acta.

[31] Kong E, Peng S, Chandra G, Sarkar C, Zhang Z, Bagh MB (2013). Dynamic palmitoylation links cytosol-membrane shuttling of acyl-protein thioesterase-1 and acyl-protein thioesterase-2 with that of proto-oncogene H-ras product and growth-associated protein-43. J Biol Chem.

[32] Hu G, Wei Y, Kang Y (2009). The multifaceted role of MTDH/AEG-1 in cancer progression. Clin Cancer Res.

[33] Yoo BK, Emdad L, Lee SG, Su ZZ, Santhekadur P, Chen D (2011). Astrocyte elevated gene-1 (AEG-1): A multifunctional regulator of normal and abnormal physiology. Pharmacol Ther.

[34] Zhang Q, He Y, Luo N, Patel SJ, Han Y, Gao R (2019). Landscape and dynamics of single immune cells in hepatocellular carcinoma. Cell.

[35] Brown ZJ, Heinrich B, Greten TF (2018). Mouse models of hepatocellular carcinoma: an overview and highlights for immunotherapy research. Nat Rev Gastroenterol Hepatol.

[36] Meng X, Zhu D, Yang S, Wang X, Xiong Z, Zhang Y (2012). Cytoplasmic metadherin (MTDH) provides survival advantage under conditions of stress by acting as RNA-binding protein. J Biol Chem.

[37] Yoo BK, Santhekadur PK, Gredler R, Chen D, Emdad L, Bhutia S (2011). Increased RNA-induced silencing complex (RISC) activity contributes to hepatocellular carcinoma. Hepatology.

[38] Roberts BJ, Svoboda RA, Overmiller AM, Lewis JD, Kowalczyk AP, Mahoney MG (2016). Palmitoylation of desmoglein 2 is a regulator of assembly dynamics and protein turnover. J Biol Chem.

[39] Yeste-Velasco M, Linder ME, Lu YJ (2015). Protein S-palmitoylation and cancer. Biochim Biophys Acta.

[40] Resh MD (2017). Palmitoylation of proteins in cancer. Biochem Soc Trans.

[41] Ko PJ, Dixon SJ (2018). Protein palmitoylation and cancer. EMBO Rep.

[42] Heakal Y, Woll MP, Fox T, Seaton K, Levenson R, Kester M (2011). Neurotensin receptor-1 inducible palmitoylation is required for efficient receptor-mediated mitogenic-signaling within structured membrane microdomains. Cancer Biol Ther.

[43] Yang Y, Hsu JM, Sun LL, Chan LC, Li CW, Hsu JL (2019). Palmitoylation stabilizes PD-L1 to promote breast tumor growth. Cell Res.

[44] Yang X, Guo Z, Sun F, Li W, Alfano A, Shimelis H (2011). Novel membrane-associated androgen receptor splice variant potentiates proliferative and survival responses in prostate cancer cells. J Biol Chem.

[45] Greenlee JD, Lopez-Cavestany M, Ortiz-Otero N, Liu K, Subramanian T, Cagir B (2021). Oxaliplatin resistance in colorectal cancer enhances TRAIL sensitivity via death receptor 4 upregulation and lipid raft localization. Elife.

[46] Cuiffo B, Ren R (2010). Palmitoylation of oncogenic NRAS is essential for leukemogenesis. Blood.

[47] Sada R, Kimura H, Fukata Y, Fukata M, Yamamoto H, Kikuchi A (2019). Dynamic palmitoylation controls the microdomain localization of the DKK1 receptors CKAP4 and LRP6. Science signaling.

[48] Kharbanda A, Walter DM, Gudiel AA, Schek N, Feldser DM, Witze ES (2020). Blocking EGFR palmitoylation suppresses PI3K signaling and mutant KRAS lung tumorigenesis. Science signaling.

[49] Kochanek DM, Wells DG (2013). CPEB1 regulates the expression of MTDH/AEG-1 and glioblastoma cell migration. Molecular cancer research: MCR.

[50] Liu L, Wu J, Ying Z, Chen B, Han A, Liang Y (2010). Astrocyte elevated gene-1 upregulates matrix metalloproteinase-9 and induces human glioma invasion. Cancer Res.

[51] Ding Z, Zhang Z, Jin X, Chen P, Lv F, Liu D (2019). Interaction with AEG-1 and MDM2 is associated with glioma development and progression and correlates with poor prognosis. Cell cycle (Georgetown, Tex).

[52] Yang L, He K, Yan S, Yang Y, Gao X, Zhang M (2017). Metadherin/Astrocyte elevated gene-1 positively regulates the stability and function of forkhead box M1 during tumorigenesis. Neuro Oncol.

[53] Kim NG, Gumbiner BM (2019). Cell contact and Nf2/Merlin-dependent regulation of TEAD palmitoylation and activity. Proc Natl Acad Sci USA.

[54] Abrami L, Kunz B, Iacovache I, van der Goot FG (2008). Palmitoylation and ubiquitination regulate exit of the Wnt signaling protein LRP6 from the endoplasmic reticulum. Proc Natl Acad Sci USA.

[55] Yount JS, Karssemeijer RA, Hang HC (2012). S-palmitoylation and ubiquitination differentially regulate interferon-induced transmembrane protein 3 (IFITM3)-mediated resistance to influenza virus. J Biol Chem.

[56] Kong C, Lange JJ, Samovski D, Su X, Liu J, Sundaresan S (2013). Ubiquitination and degradation of the hominoid-specific oncoprotein TBC1D3 is regulated by protein palmitoylation. Biochemical and biophysical research communications.

[57] Lakkaraju AK, Abrami L, Lemmin T, Blaskovic S, Kunz B, Kihara A (2012). Palmitoylated calnexin is a key component of the ribosome-translocon complex. EMBO J.

[58] Fredericks GJ, Hoffmann FW, Rose AH, Osterheld HJ, Hess FM, Mercier F (2014). Stable expression and function of the inositol 1,4,5-triphosphate receptor requires palmitoylation by a DHHC6/selenoprotein K complex. Proc Natl Acad Sci USA.

[59] Fairbank M, Huang K, El-Husseini A, Nabi IR (2012). RING finger palmitoylation of the endoplasmic reticulum Gp78 E3 ubiquitin ligase. FEBS letters.

[60] Qiu N, Abegg D, Guidi M, Gilmore K, Seeberger PH, Adibekian A (2021). Artemisinin inhibits NRas palmitoylation by targeting the protein acyltransferase ZDHHC6. Cell Chem Biol.

[61] Wang F, Pang JD, Huang LL, Wang R, Li D, Sun K (2018). Nanoscale polysaccharide derivative as an AEG-1 siRNA carrier for effective osteosarcoma therapy. International journal of nanomedicine.

[62] Li P, He Y, Chen T, Choy KY, Chow TS, Wong ILK (2021). Disruption of SND1-MTDH interaction by a high affinity peptide results in SND1 degradation and cytotoxicity to breast cancer cells *in vitro* and *in vivo*. Molecular cancer therapeutics.

