# Supplementary material for: The palmitoylation of AEG-1 dynamically modulates the progression of hepatocellular carcinoma
Source: Theranostics. 2022 Oct 3;12(16):6898–914. doi: 10.7150/thno.78377 (PMC9576614; doi:10.7150/thno.78377)
Supplement: Supplementary file 1 — Supplementary figures and tables. [file thnov12p6898s1.pdf]

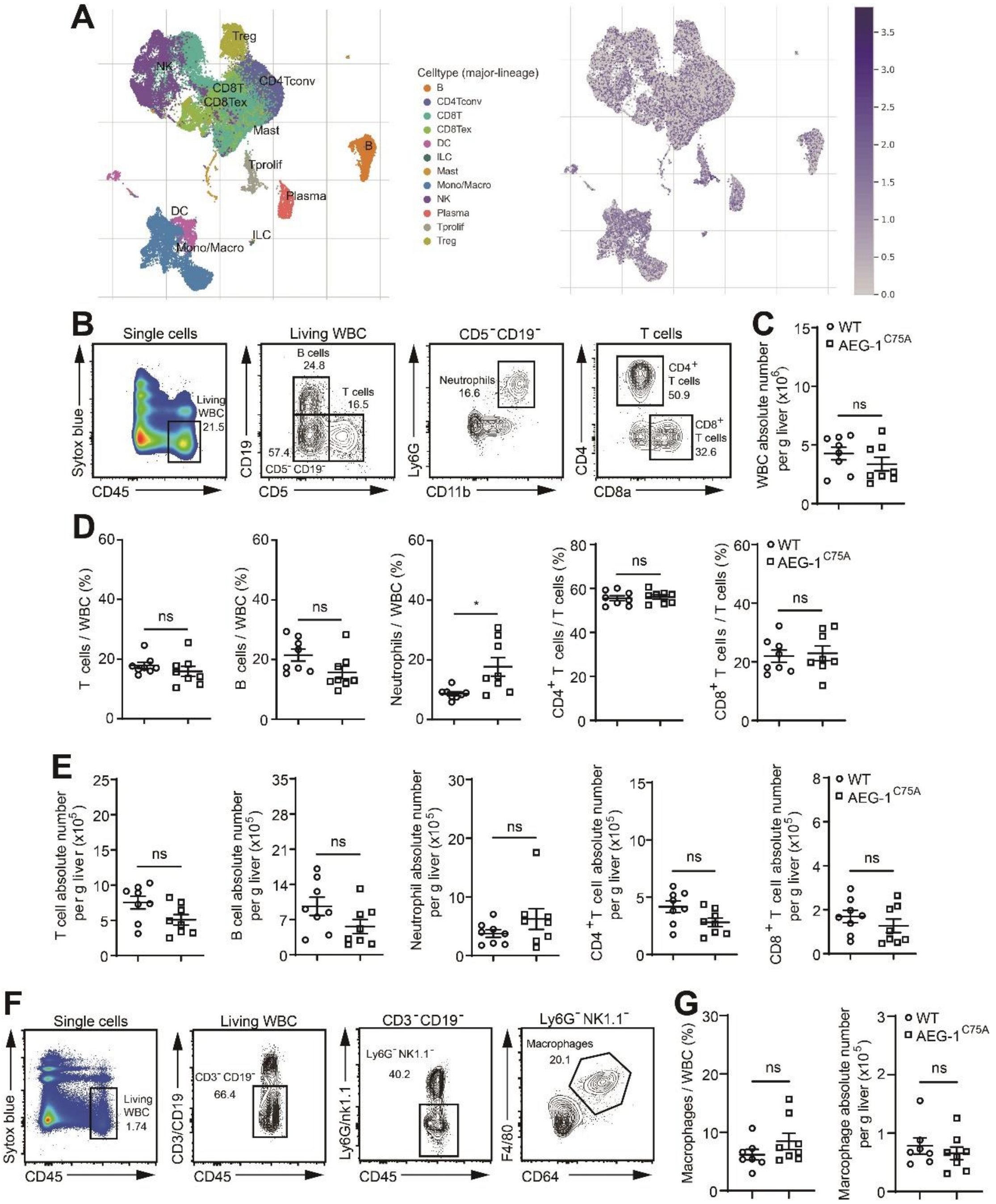

Supplementary Figure 1. The mutation of AEG-1 palmitoylation site does not affect lymphocytes, neutrophils and macrophages in mice under normal feeding conditions. (A) The expression levels of AEG-1 in different immune cells were analyzed using the single-cell sequencing database TISCH. **(B)** Different immune cells gating strategy in mouse liver. **(C)** Absolute number of WBC in mouse livers from WT and AEG-1^{C75A} were quantified by Attune NxT volumetric flow cytometer. **(D, E)** Comparison of different immune cell frequencies in WBC and absolute number in the livers of WT and AEG-1^{C75A} mice. **(F)** Macrophage gating strategy in mouse liver. **(G)** Comparison of macrophage frequency in WBC and absolute number in the livers of WT and AEG-1^{C75A} mice (n=8). The data are presented as the mean ± SEM. **p* < 0.05, ns: no significance. Statistical significance was determined by unpaired two-tailed Student's t-test for comparison between two groups.

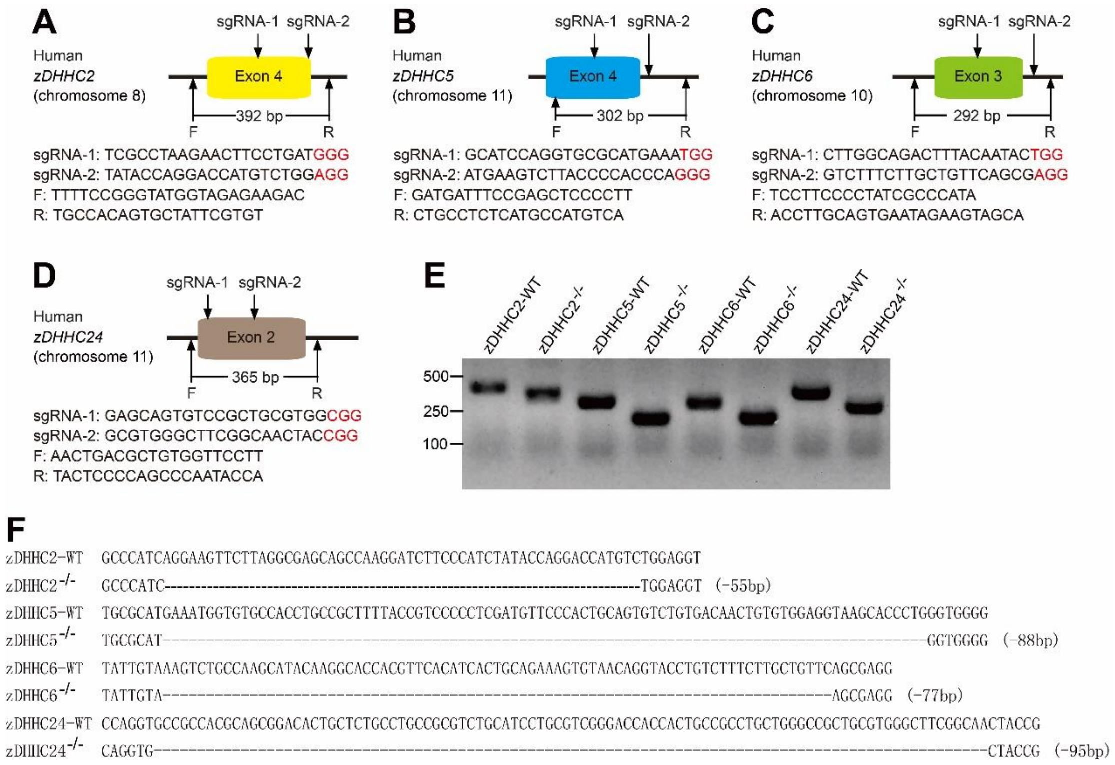

Supplementary Figure 2. Generation of HEK293T-zDHHCs knockout cell lines. (A-D) Schematic diagram of the small guide RNA (sgRNA) sequences used to target exons of four zDHHCs gene, respectively. Protospacer adjacent motifs (PAMs) are shown in red letters, and PCR detection primers are listed below the sgRNA sequences. **(E, F)** Validation of HEK293T-zDHHCs knockout cell lines by agarose gel electrophoresis **(E)** and sequencing **(F)**.

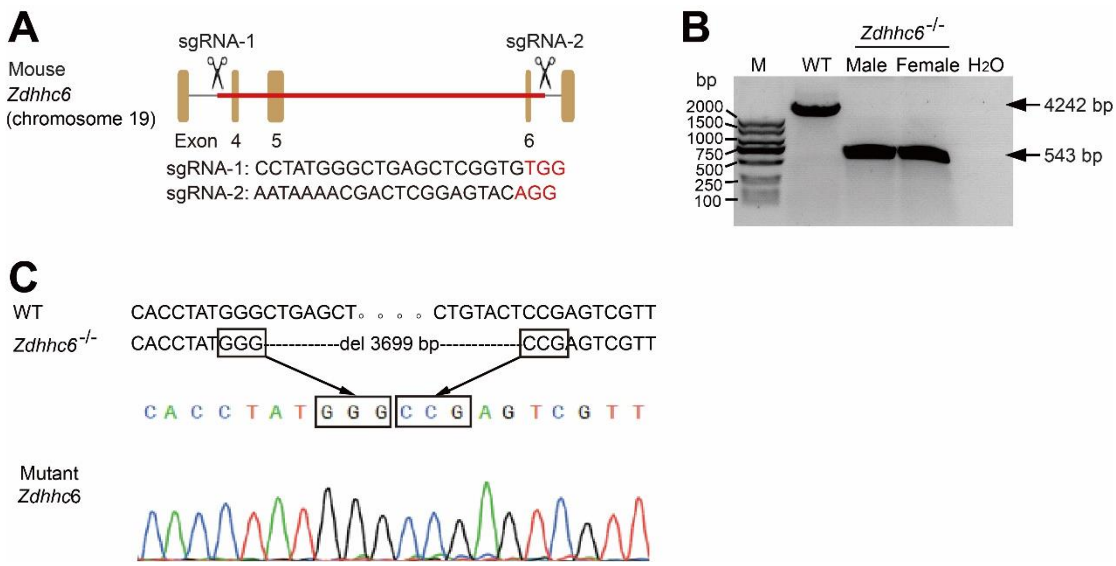

Supplementary Figure 3. Generation of Zdhhc6$^{-/-}$ mice. (A) Schematic diagram of two sgRNA sequences used to target intron 1 and 4 of Zdhhc6 gene. PAMs are shown in red letters. **(B, C)** Validation of Zdhhc6$^{-/-}$ mouse by agarose gel electrophoresis **(B)** and sequencing **(C)**.

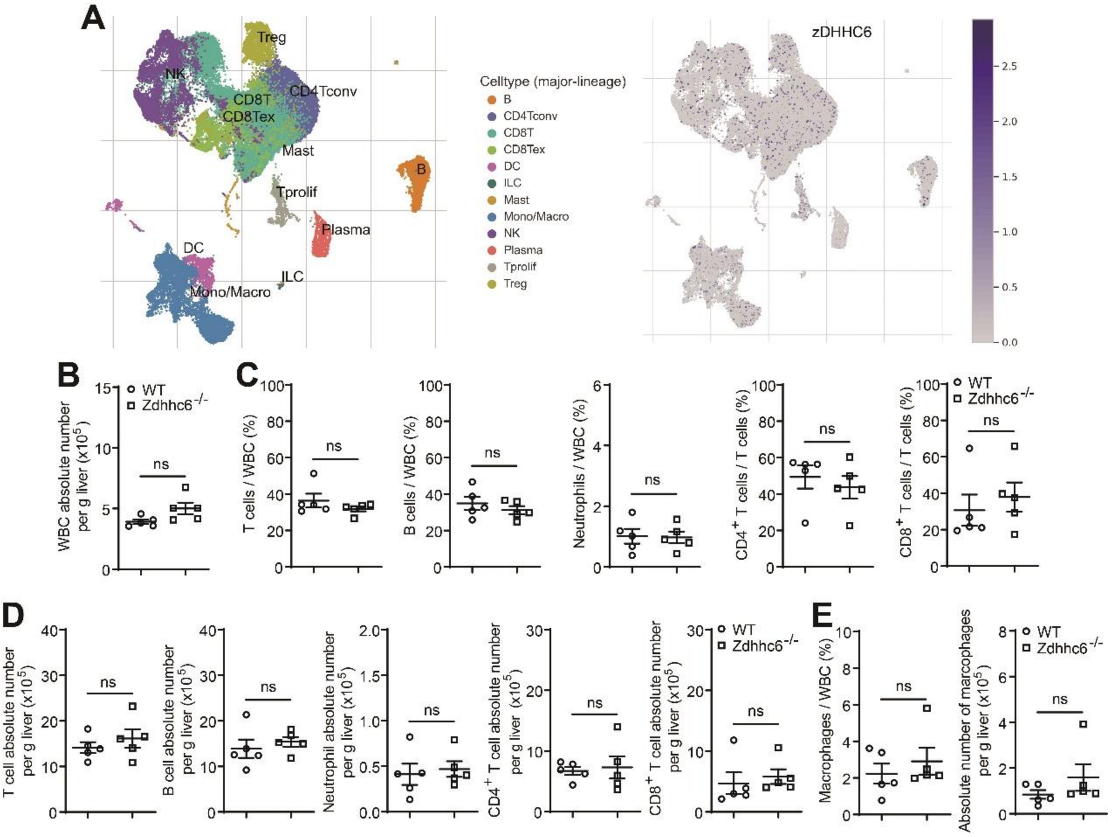

Supplementary Figure 4. Knockout of Zdhhc6 does not affect lymphocytes, neutrophils and macrophages in mice under normal feeding conditions. (A) The expression levels of Zdhhc6 in different immune cells were analyzed using the single-cell sequencing database TISCH. **(B)** Absolute number of WBC in mouse livers from WT and Zdhhc6$^{-/-}$ were quantified by Attune NxT volumetric flow cytometer. **(C, D)** Comparison of different immune cell frequencies in WBC and absolute number in the livers of WT and Zdhhc6$^{-/-}$ mice. **(E)** Comparison of macrophage frequency in WBC and absolute number in the livers of WT and Zdhhc6$^{-/-}$ mice (n=5). The data are presented as the mean ± SEM. Ns: no significance. Statistical significance was determined by unpaired two-tailed Student's t-test for comparison between two groups.

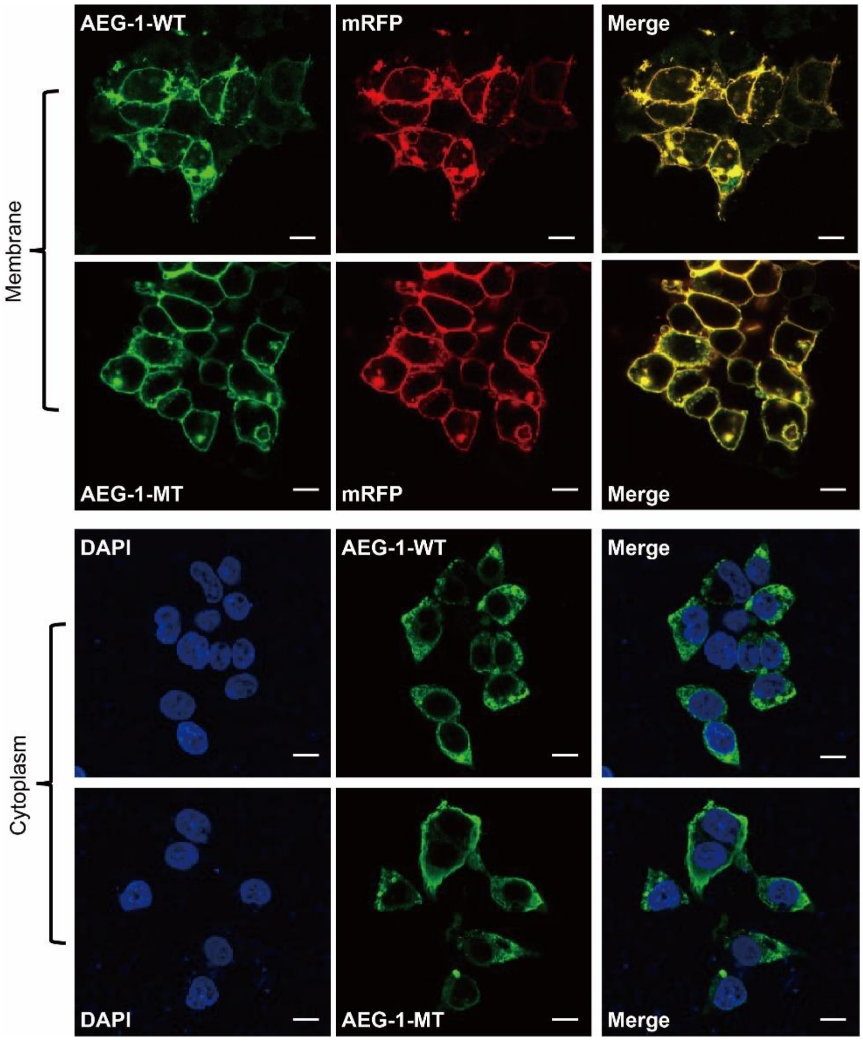

Supplementary Figure 5. Loss of palmitoylation does not affect the subcellular localization of AEG-1. AEG-1-WT and AEG-1-MT were overexpressed and immunostained for the detection of the subcellular localization of AEG-1-WT and AEG-1-MT in Huh7 cells. mRFP as a membrane marker. Nuclei were stained with DAPI.

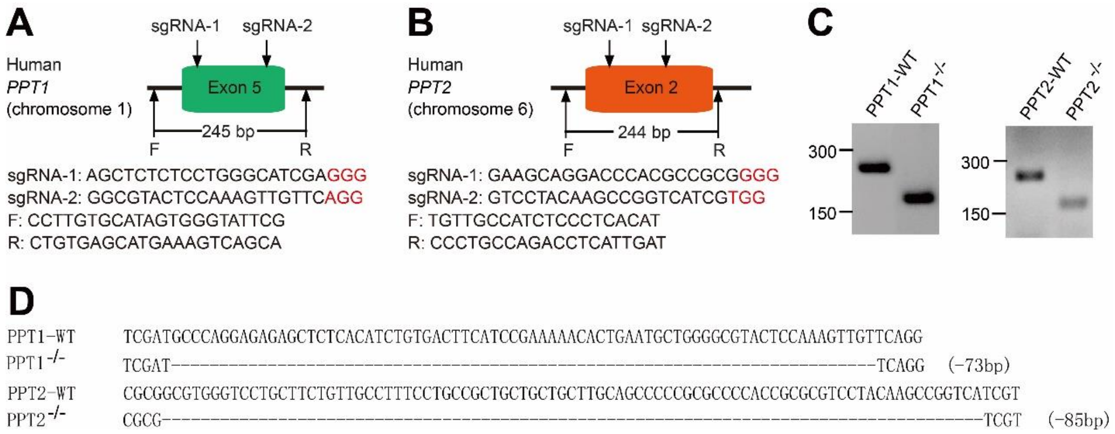

Supplementary Figure 6. Generation of PPT1 and PPT2 knockout cell lines. (A, B) Schematic representation of the sgRNA sequences used to target exons of PPT1 and PPT2 genes, respectively. PAMs are shown in red letters, and PCR detection primers are below the sgRNA sequences. **(C, D)** Validation of HEK293T-PPT1$^{-/-}$ and HEK293T-PPT2$^{-/-}$ cell lines by agarose gel electrophoresis **(C)** and sequencing **(D)**.

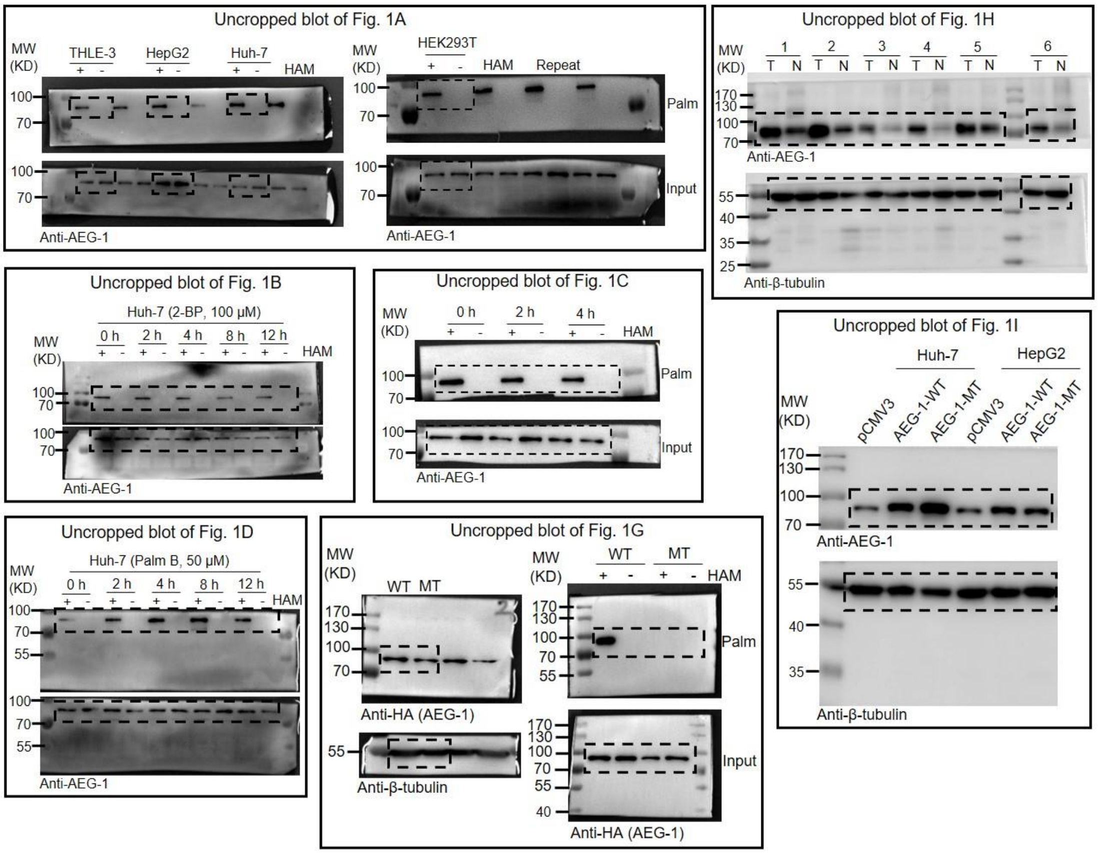

789

790 **Supplementary Figure 7. Uncropped blot 1.**

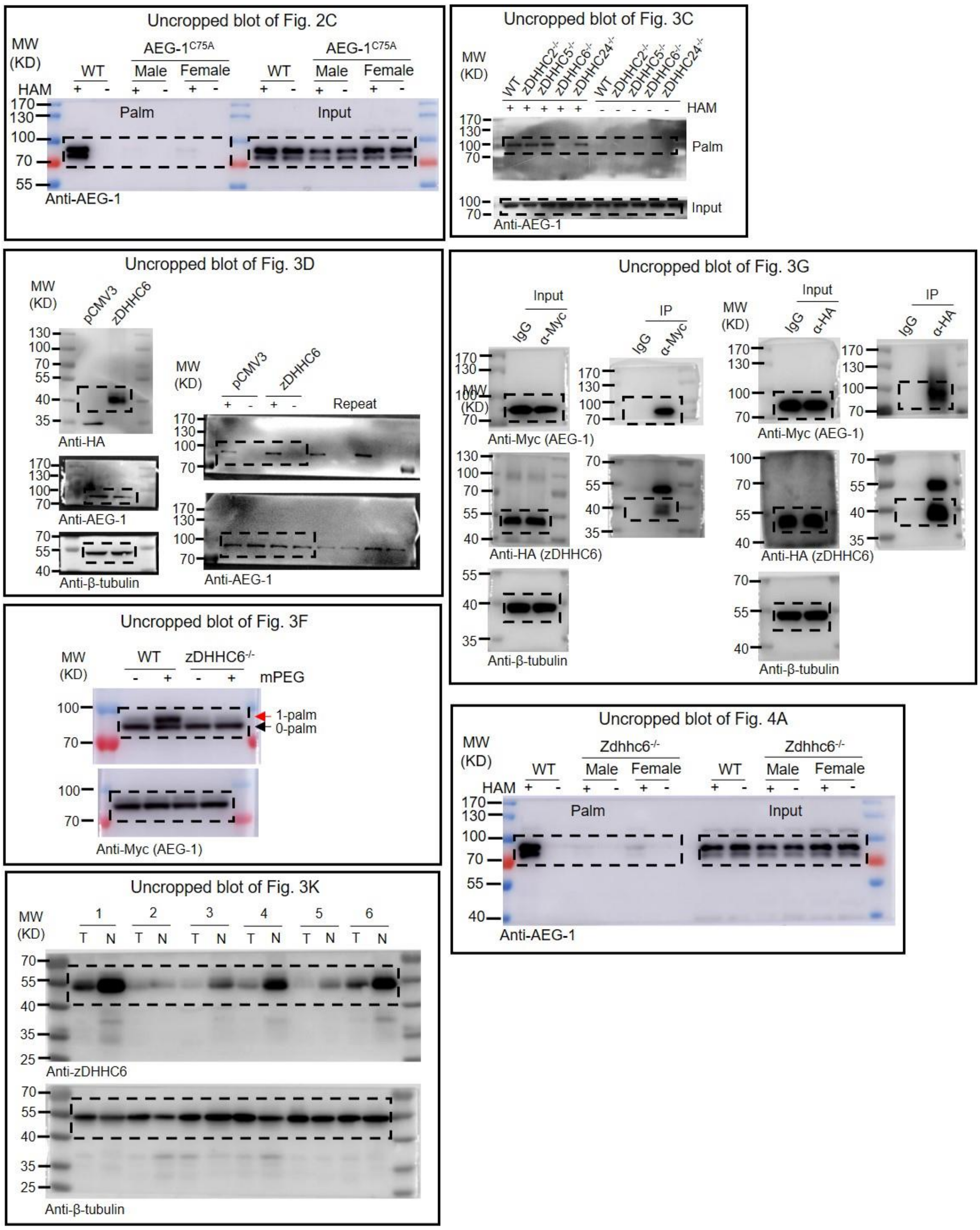

Supplementary Figure 8. Uncropped blot 2.

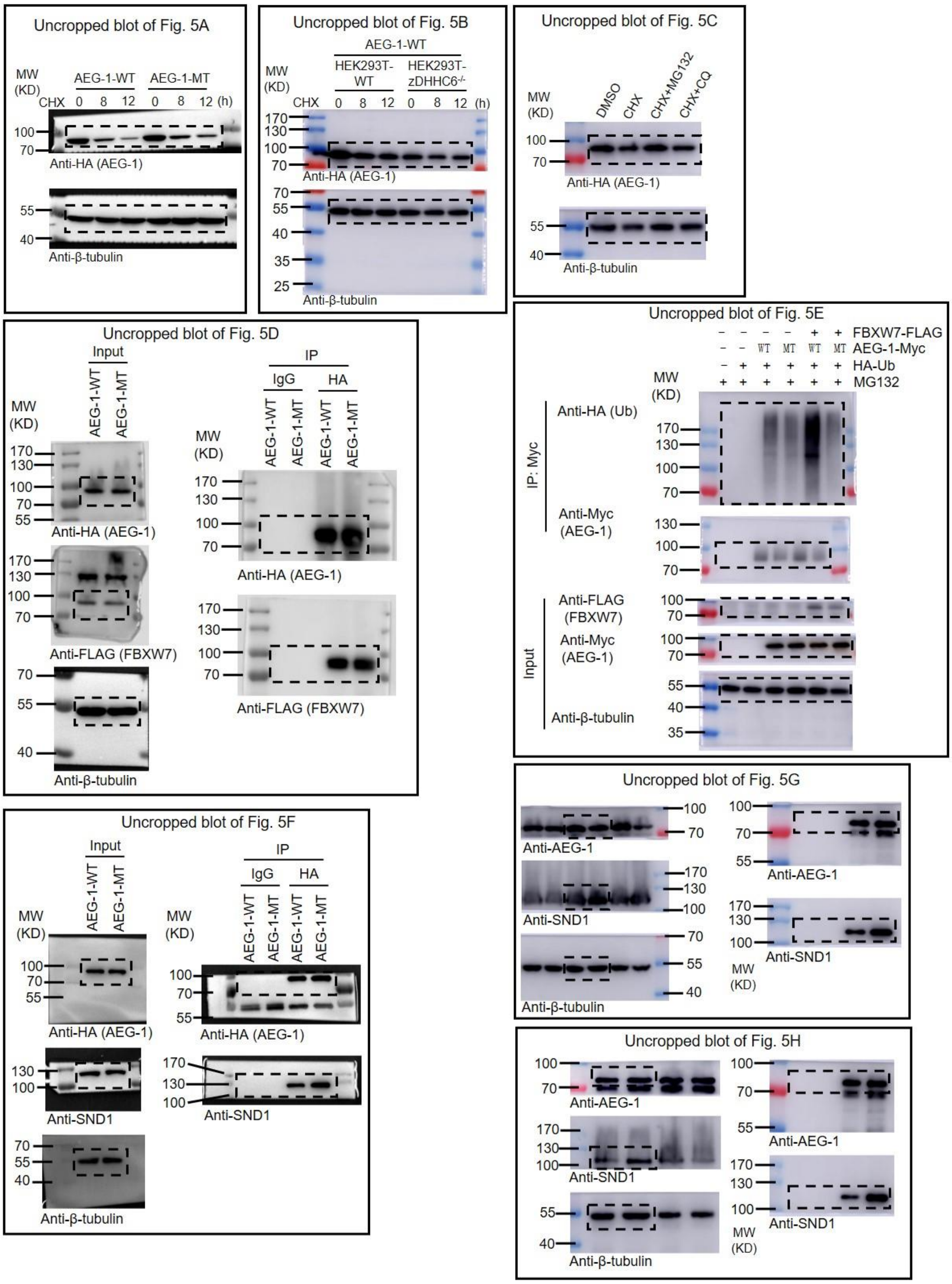

Supplementary Figure 9. Uncropped blot 3.

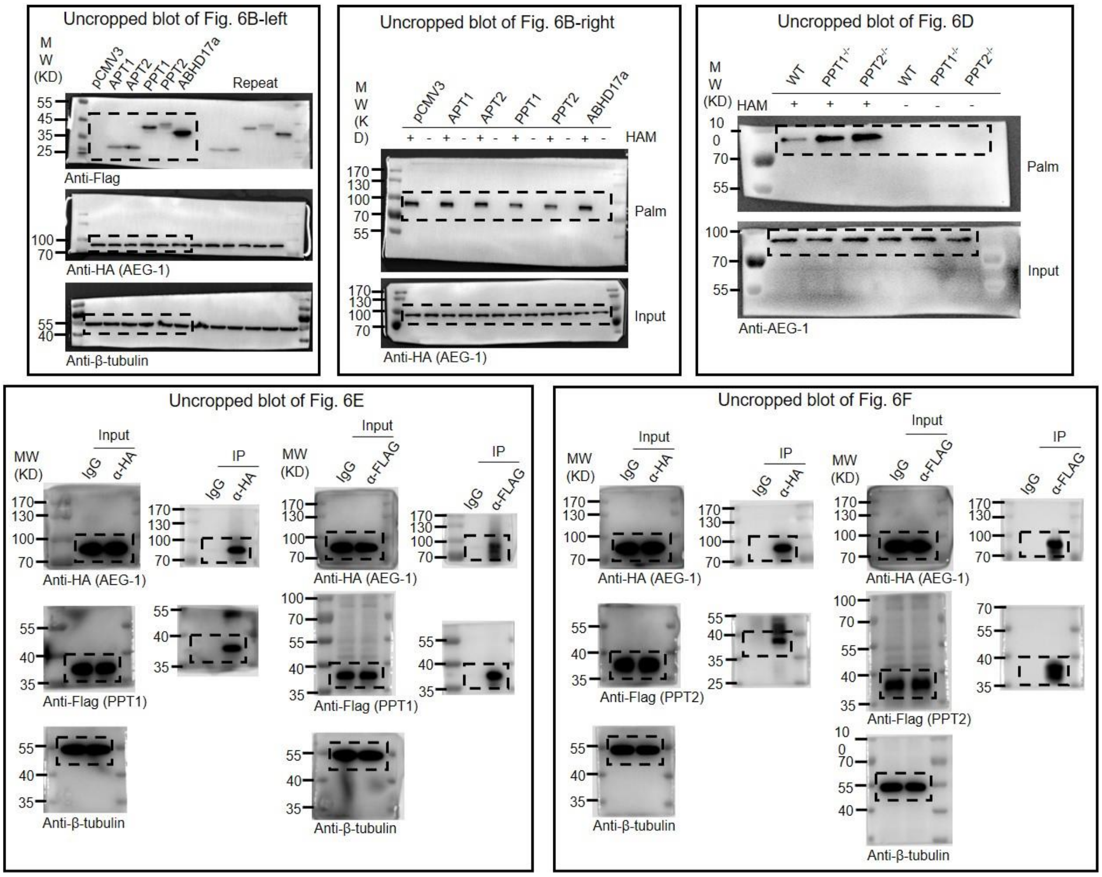

Supplementary Figure 10. Uncropped blot 4.

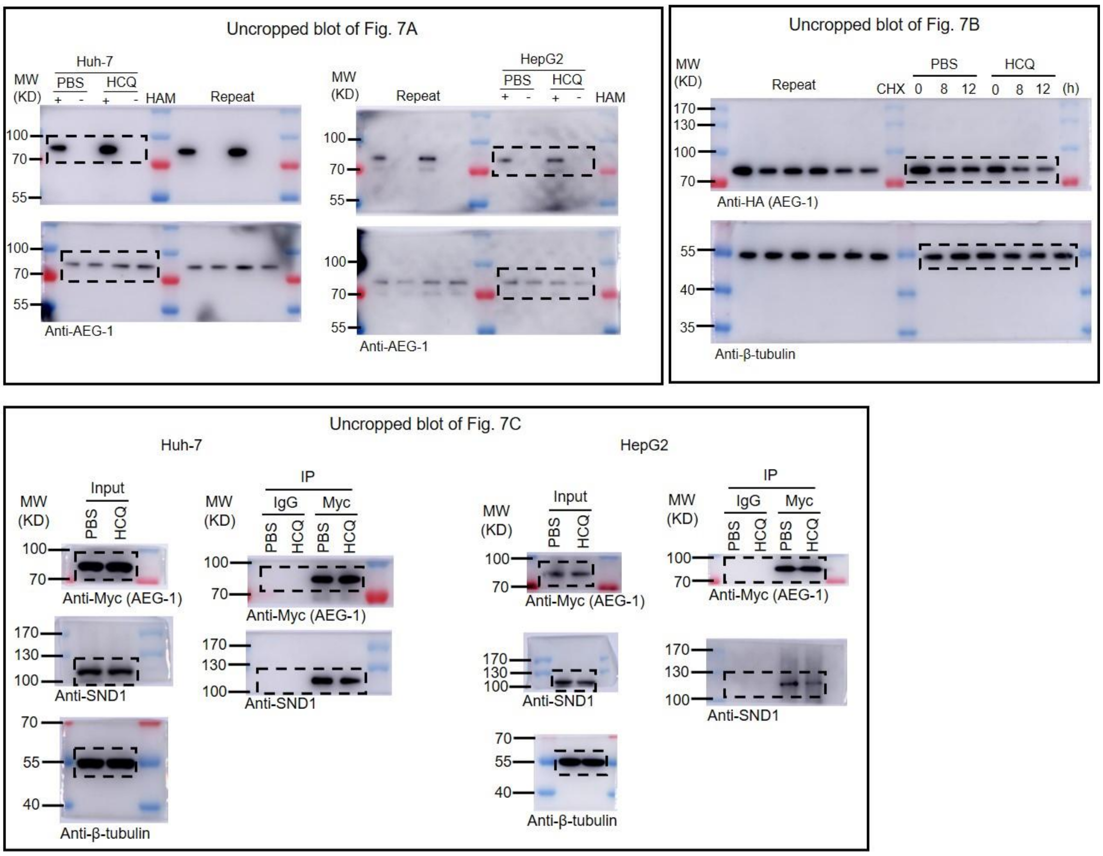

Supplementary Figure 11. Uncropped blot 5.

Supplementary Tables:

Supplementary Table 1. Primer pairs of gRNA used for generation of PPT1, PPT2, zDHHC2, zDHHC5, zDHHC6 and zDHHC24 knockout cell lines

Gene name (human)	Sequence (5' to 3')
PPT1 gRNA #1	CACCGAGCTCTCTCCTGGGCATCGA (F)
	AAACTCGATGCCCAGGAGAGAGCTC (R)
PPT1 gRNA #2	CACCGGCGTACTCCAAAGTTGTTC (F)
	AAACGAACAACTTTGGAGTACGCC (R)
PPT2 gRNA #1	CACCGAAGCAGGACCCACGCCGCG (F)
	AAACCGCGGCGTGGGTCCTGCTTC (R)
PPT2 gRNA #2	CACCGTCCTACAAGCCGGTCATCG (F)
	AAACCGATGACCGGCTTGTAGGAC (R)
zDHHC2 gRNA #1	CACCGTCGCCTAAGAACTTCCTGAT (F)
	AAACATCAGGAAGTTCTTAGGCGAC (R)
zDHHC2 gRNA #2	CACCGTATACCAGGACCATGTCTGG (F)
	AAACCCAGACATGGTCCTGGTATAC (R)
zDHHC5 gRNA #1	CACCGCATGGAGGTGCGCATGAAA (F)
	AAACTTTCATGCGCACCTGGATGC (R)
zDHHC5 gRNA #2	CACCGATGAAGTCTTACCCCACCCA (F)
	AAACTGGGTGGGGTAAGACTTCATC (R)
zDHHC6 gRNA #1	CACCGCTTGGCAGACTTTACAATAC (F)
	AAACGTATTGTAAAGTCTGCCAAGC (R)
zDHHC6 gRNA #2	CACCGTCTTTCTTGCTGTTCAGCG (F)
	AAACCGCTGAACAGCAAGAAAGAC (R)
zDHHC24 gRNA #1	CACCGAGCAGTGTCCGCTGCGTGG (F)
	AAACCCACGCAGCGGACACTGCTC (R)
zDHHC24 gRNA #2	CACCGCGTGGGCTTCGGCAACTAC (F)
	AAACGTAGTTGCCGAAGCCCACGC (R)

Supplementary Table 2. Primer pairs used for PCR analysis

Gene name (human)	Sequence (5’ to 3’)	Usage
PPT1-genome	CCTTGTGCATAGTGGGTATTCG (F)<br>CTGTGAGCATGAAAGTCAGCA (R)	Genomic PCR detection for knockout cell lines
PPT2-genome	TGTTGCCATCTCCCTCACAT (F)<br>CCCTGCCAGACCTCATTGAT (R)	
zDHHC2-genome	TTTTCCGGGTATGGTAGAGAAGAC (F)<br>TGCCACAGTGCTATTCGTGT (R)	
zDHHC5-genome	GATGATTTCCGAGCTCCCCTT (F)<br>CTGCCTCTCATGCCATGTCA (R)	
zDHHC6-genome	TCCTTCCCCTATCGCCCATA (F)<br>ACCTTGCAGTGAATAGAAGTAGCA (R)	
zDHHC24-genome	AACTGACGCTGTGGTTCCTT (F)<br>TACTCCCCAGCCCAATACCA (R)	

Supplementary Table 3. Primer pairs used for qRT-PCR analysis

Gene name	Sequence (5' to 3')	Usage
zDHHC1	CCGCGGCGCGCAGCTGTTT (F)<br>GGGCCGTCTTGTTGGAGGGCT (R)	qPCR detection for endogenous gene expression levels in human cell lines
zDHHC2	CCCTCCTGCTCGGCTGGTCCT (F)<br>AGGCCATCAGGCACACAACTTGTTC (R)	
zDHHC3	GCTGACGGACCCCGTGAGAACCT (F)<br>GGCACTTGCTGCGGCCTGAA (R)	
zDHHC4	CTGGCGGTAAGGCCGCCTCC (F)<br>GCGGCCCTACACACAAGGGGA (R)	
zDHHC5	GCTTCTCTGAGGCAGGACGGCAC (F)<br>ACTGTGTCTCACCCGCGAGCCC (R)	
zDHHC6	CCCTCTGGGGTGGAAACCGGAAAT (F)<br>CTGCAGTGATGTGAACGTGGTGCC (R)	
zDHHC7	TGACTGCCGACCATCTGCCTGC (F)<br>ACTGCCCCAAGCAGGAGGCTTT (R)	
zDHHC8	AGCCCTACACGGCACACGCT (F)<br>CGTGGCACCATGGCGGATGTGT (R)	
zDHHC9	GGTCGCTGCTTCCCGACGGA (F)<br>TGCTAGCCCTGGAAGACAATCTCCT (R)	
zDHHC11	CAGGGGAACTGATGGGCGGTCG (F)<br>GACCCCGGCCAGAGCCGAGT (R)	
zDHHC12	TGGCATGGTCAGGCCTCCGGTT (F)<br>GAGCAGGCTGGCCACCAACGA (R)	
zDHHC13	CGCCAGCAGGAAGTGGGAGA (F)<br>CATCTCCCCAGGAGCCCACA (R)	
zDHHC14	CCGGCAGACGGGCGTCTTCTAC (F)<br>AGGTACGGACAGTCGAAGGCGAAG (R)	

Continued

Gene name	Sequence (5’ to 3’)	Usage
zDHHC15	ACAGGGTGGGGAGGAGGCAGAA (F)<br>CGGAAGGGTGATCCGTCAGGCA (R)	
zDHHC16	GTCGGCAGGCGGCAGGTCTT (F)<br>GCTGCTCCACTGCAGGGATGGT (R)	
zDHHC17	TGCCCCTTCTCCACCCAGAGGAAA (F)<br>GCCTTGACTATGTCCCATGTGCTGT (R)	
zDHHC18	GGCAACTGTGTGGGGAGACGGA (F)<br>TCCCTGAGCGCGCAACGTCA (R)	
zDHHC19	ACCAGGGCTGTGCCAGCAAC (F)<br>CAGGCCCCACCACTCTCTGC (R)	
zDHHC20	GGCGGCGCCTCGGACTTTTG (F)<br>CGGCGACGGTGACTCGGACG (R)	qPCR detection for endogenous gene expression levels in human cell lines
zDHHC21	AGCCAGCGGCGACGAATGAA (F)<br>ACTGGCAAAGCAGCGCCTCCAA (R)	
zDHHC22	CGCCTCAAATGAAAACGCTCTTCGC (F)<br>TACATTCCTGTGTCAGCGGGTCCT (R)	
zDHHC23	CTCCGGGGAAGCCGACCCAT (F)<br>AACCTCCAACGCCTGCCCTCT (R)	
zDHHC24	CACTGTCGGCCCTGCTGCGA (F)<br>GTGCCAGAGACACTCTGCCTGTGA (R)	
APT1	TCAACCCCGCTGCCCGCCAT (F)<br>GCCCATCCGTGCCCAGTATCTCC (R)	
APT2	TGCTGCCACCGTGTCTGGAG (F)<br>GGATGGTGGAGAGGGCGTCA (R)	
PPT1	ACCTGCGCTTCTCGGGCGCT (F)<br>ATTGCAACAGCTGTCTCCCATCCCA (R)	

Continued

Gene name	Sequence (5' to 3')	Usage
PPT2	CCGCGTTGTTCATGGCTGAGGCG (F)<br>CCCAGCATGCTCCCGCCAAC (R)	qPCR detection for endogenous gene expression levels in human cell lines
ABHD17a	TGCGCACCAGGTACGGCATCAG (F)<br>CAGCACCACCGCGGCACACT (R)	
18S	AGGGCACCACCAGGAGTGGA (F)<br>CCACCACCCACGGAATCGAG (R)	Reference gene in human
Pten	GCCAACCGATACTTCTCTCCA (F)<br>GGATCAGAGTCAGTGGTGTCA (R)	qPCR detection for tumor suppressor genes in mouse liver
Cdkn1a	GGACAAGAGGCCCAGTACTTC (F)<br>CTGCGCTTGGAGTGATAGAAA (R)	
Spry2	CGGAGCAGTACAAGGACAAGT (F)<br>TGTAGGCATGCAGACCCAAAT (R)	
Tgfbr2	TGGAGAGCATGAAAGACAGTGT (F)<br>CAGCACTCGGTCAAAGTCTCA (R)	
Hprt	CGTCGTGATTAGCGATGATG (F)<br>ACAGAGGGCCACAATGTGAT (R)	Reference gene in mouse
PTEN	GCGTGCAGATAATGACAAGGA (F)<br>TGCTAGCCTCTGGATTTGACG (R)	qPCR detection for tumor suppressor genes in HepG2 cells
CDKN1A	TGTCTTGTACCCTTGTGCCTC (F)<br>CGGCGTTTGGAGTGGTAGAAA (R)	
SPRY2	CAACTGTGCTGACAACCCATG (F)<br>GGCCTGTTAACCCGGTCATAA (R)	
TGFBR2	AACGTGTTGAGAGATCGAGGG (F)<br>CAGCACTCAGTCAACGTCTCA (R)	
GAPDH	CAACAGCGACACCCACTCCT (F)<br>CACCCTGTTGCTGTAGCCAAA (R)	Reference gene in human

Supplementary Table 4. Primer pairs used for generation of Zdhhc6$^{-/-}$ and Aeg-1 point mutation mice

Gene name (mouse)	Sequence (5’ to 3’)
Zdhhc6 gRNA #1	CACCGCCTATGGGCTGAGCTCGGTG (F)<br>AAACCACCGAGCTCAGCCCATAGG (R)
Zdhhc6 gRNA #2	CACCGAATAAAACGACTCGGAGTAC (F)<br>AAACGTACTCCGAGTCGTTTTATT (R)
Aeg-1 point mutation gRNA #1	CACCGGCTGGGCCGCGGCTTGCGC (F)<br>AAACGCGCAAGCCGCGGCCCAGCC (R)
Aeg-1 point mutation gRNA #2	CACCGCGGGCGCCGGCGCAAGCCG (F)<br>AAACCGGCTTGCGCCGGCGCCCGC (R)
Zdhhc6-genome	ATTGATTGTGATGTTGACTAGCTTC (F)<br>CAGAAGTAGGTGTGACATTCTAAC (R)
Aeg-1-genome	CTCCCACGACTGTTCCAGCGG (F)<br>AACCTCCACATGCAGCCTACTTTCT (R)
